#  Increasing the resolution of malaria early warning systems for use by local health actors

**DOI:** 10.1186/s12936-025-05266-0

**Published:** 2025-01-30

**Authors:** Michelle V. Evans, Felana A. Ihantamalala, Mauricianot Randriamihaja, Vincent Herbreteau, Christophe Révillion, Thibault Catry, Eric Delaitre, Matthew H. Bonds, Benjamin Roche, Ezra Mitsinjoniala, Fiainamirindra A. Ralaivavikoa, Bénédicte Razafinjato, Oméga Raobela, Andres Garchitorena

**Affiliations:** 1 MIVEGEC, Univ. Montpellier, CNRS, IRD, Montpellier, France; 2 NGO Pivot, Ranomafana, Ifanadiana Madagascar; 3 Department of Global Health and Social Medicine, Blavatnik Institute at Harvard Medical School, Boston, MA USA; 4 Espace-Dev, IRD, Univ. Montpellier, Univ. Antilles, Univ. Guyane, Univ Réunion, Univ Nouvelle-Calédonie, Montpellier, France; 5 Espace-Dev, Université de La Réunion, Saint Denis, La Réunion, France; 6 National Malaria Programme, Ministry of Health, Antananarivo, Madagascar

**Keywords:** Malaria, Disease forecasting, Climate, Digital health, Precision public health

## Abstract

**Background:**

The increasing availability of electronic health system data and remotely-sensed environmental variables has led to the emergence of statistical models capable of producing malaria forecasts. Many of these models have been operationalized into malaria early warning systems (MEWSs), which provide predictions of malaria dynamics several months in advance at national and regional levels. However, MEWSs rarely produce predictions at the village-level, the operational scale of community health systems and the first point of contact for the majority of rural populations in malaria-endemic countries.

**Methods:**

This study developed a hyper-local MEWS for use within a health-system strengthening intervention in rural Madagascar. It combined bias-corrected, village-level case notification data with remotely sensed environmental variables at spatial scales as fine as a 10 m resolution. A spatio-temporal hierarchical generalized linear regression model was trained on monthly malaria case data from 195 communities from 2017 to 2020 and evaluated via cross-validation. The model was then integrated into an automated workflow with environmental data updated monthly to create a continuously updating MEWS capable of predicting malaria cases up to three months in advance at the village-level. Predictions were transformed into indicators relevant to health system actors by estimating the quantities of medical supplies required at each health clinic and the number of cases remaining untreated at the community level.

**Results:**

The statistical model was able to accurately reproduce village-level case data, performing nearly five times as well as a null model during cross-validation. The dynamic environmental variables, particularly those associated with standing water and rice field dynamics, were strongly associated with malaria incidence, allowing the model to accurately predict future incidence rates. The MEWS represented an improvement of over 50% compared to existing stock order quantification methods when applied retrospectively.

**Conclusion:**

This study demonstrates the feasibility of developing an automatic, hyper-local MEWS leveraging remotely-sensed environmental data at fine spatial scales. As health system data become increasingly digitized, this method can be easily applied to other regions and be updated with near real-time health data to further increase performance.

**Supplementary Information:**

The online version contains supplementary material available at 10.1186/s12936-025-05266-0.

## Background

Data systems play a key role in malaria control initiatives. Indeed, malaria surveillance is one of three pillars of the World Health Organization (WHO) Global Technical Strategy for Malaria 2016–2030 [1]The strategy stresses the need to strengthen local health management information systems (HMISs) to better track progress towards elimination and heterogeneity within a country. In addition to surveillance, malaria early warning systems (MEWSs), which use statistical and mathematical models to forecast malaria dynamics up to several months in advance as a function of environmental variables, can aid health systems in preventing malaria outbreaks and improving system reactivity [2, 3].Because malaria is a vector-borne pathogen, it is sensitive to environmental variables, particularly climatic variables such as temperature and precipitation [4], facilitating the creation of accurate early warning systems [5]. Relevant environmental variables, such as temperature, precipitation, and vegetation indices, can be derived from satellite imagery, whose resolution, frequency, and accessibility are continuously improving [6]. With the evolving capabilities of health and environmental data systems, it is increasingly feasible to link these two data systems and create disease forecasts. However, while several MEWSs have been developed at global, regional, and national scales, the routine integration of environmental data in MEWSs remains rare [7].

The spatial scale at which MEWSs are developed determines the potential users of the tool for decision-making. While current MEWSs can effectively inform international organizations and national programme managers, few MEWSs have been developed at local scales (< 1km^2^) relevant for operational use by health actors implementing malaria control activities within a health district. Combined with increased ownership over local budgets and policies, increasing the resolution of MEWSs could allow district managers and medical teams to adapt interventions to the village or local level, such as hotspot targeting, last mile delivery, and community health worker (CHW) programmes. Malaria hotspots (zones with consistently high incidence rates) have been proposed as an appropriate unit to target with vector control or human health interventions, such as mass drug administration [8], although the ability of these interventions to impact regions outside of hotspots is limited [9, 10]. Last mile delivery interventions support the management and distribution of medical stocks, such as anti-malarials and bed nets at the lowest scale of the health system, often the CHW or the household [11]. By delivering medical products and services to remote populations, these programmes aim to remove geographic barriers to prevention and treatment of malaria within a medically-relevant timeframe. CHW programmes provide basic health services to local communities of several hundred to several thousand people [12]. While their focus has traditionally been maternal and child care, the professionalization of CHWs and an associated expansion of their responsibilities to include non-communicable diseases, immunization, mental health, and epidemiological surveillance is being implemented in many countries [13]. In Madagascar, CHWs traditionally diagnose and treat malaria in children under 5 years of age, and recent pilot programmes have demonstrated the success of expanding responsibilities to include additional malaria interventions, such as the provision of intermittent preventive treatment to pregnant women [14] or proactive screening and treatment [15]. These programmes are generally planned and overseen by the heads of primary health clinics (PHCs), who oversee community programmes for their communes. The successes of community-targeted programmes have prompted calls for the increased development of digital health tools for programmes implemented at local scales, especially in sub-Saharan Africa [16, 17].

In order for a MEWS to be usable by programmes at the local-scale, it must not only provide predictions at a relevant spatial scale, but it should also be timely (i.e. little delay between the availability of input data and availability of predictions) and contextually relevant. This presents challenges as HMIS data are rarely reported at the scale of individual villages or communities, and when they are, tend to suffer from substantial data quality issues and biases [18–20]. In addition, the predictive variables used in the MEWS statistical model must also be at finer spatial scales than typically available, necessitating the pre- and post-processing of satellite imagery in continuous time [21]. Finally, outputs of a MEWS should be made contextually relevant by integrating predictions with existing HMIS data, such as historical case burdens, information on diagnostic and treatment supplies, and ongoing programmes. It should be noted, that even with a local, timely, contextually-relevant MEWS, broader health system interventions, such as the distribution of decision-making power to local actors and training in data literacy, are necessary for disease forecasts to translate to programmatic adjustments. In consideration of these challenges, this project developed SMALLER (‘Surveillance and control of Malaria At the Local Level using E-health platfoRms’), a hyper-local MEWS specifically for use in the context of a health-system strengthening intervention in a rural district of Madagascar.

## Methods

**Fig. 1:**
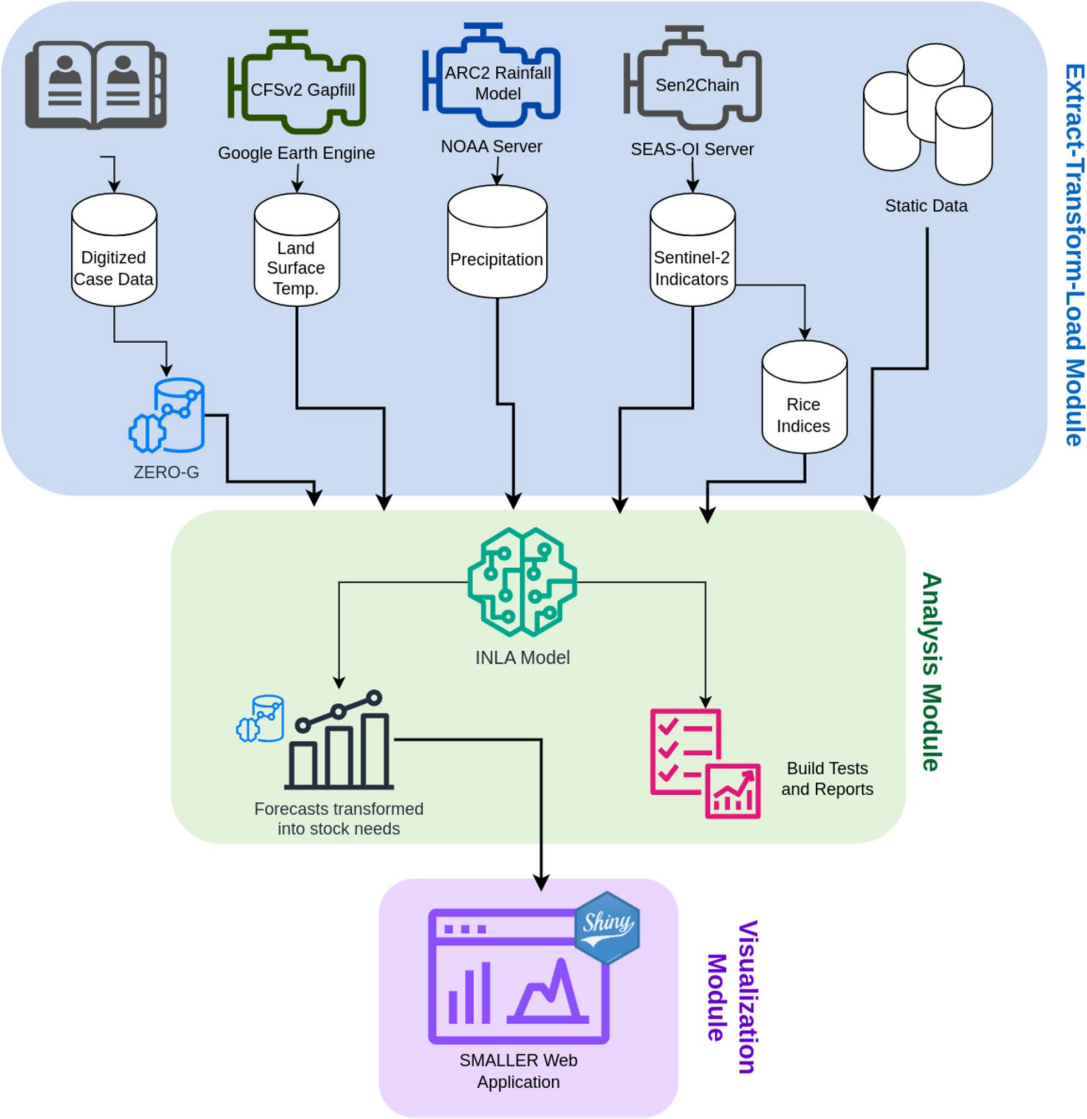
Schema of SMALLER MEWS web application architecture. The workflow is divided into three modules: Extract-Transform-Load, Analysis, and Visualization. (1) Extract-Transform-Load: Incidence data are created from digitized health register data that has been treated for under-estimation via the ZERO-G method. Temperature data are treated via the Climate Forecast System Version 2 (CFSv2) gapfill method in Google Earth Engine. Precipitation data are sourced from the Africa Rainfall Climatology Version 2 (ARC2) model housed on a NOAA server. Indicators derived from the Sentinel-2 satellite are processed via the Sen2Chain python framework on a server at Surveillance de l'Environnement Assistée par Satellite pour l'Ocean Indien (SEAS-OI) and further transformed into indicators representing rice field dynamics. (2) Analysis: Data sources are combined via an INLA model to create forecasts of reported symtomatic case rates at the community scale. Forecast case rates are back-transformed into stock needs at the PHC and cases remaining to be treated at the community health site using the ZERO-G method to account for care-seeking rates. (3) Visualization: Predictions are made accessible in an interactive dashboard via a Shiny application. The full workflow is updated on a monthly basis via a semi-automated process using the targets package

First, an overview of the MEWS workflow (Fig. 1) is provided to orient the reader before entering into a detailed discussion of each step below. HMIS data and socio-environmental variables were collected at the spatial scale of community health catchments, ensuring data quality by treating HMIS data with a zero-adjusted, gravity model estimator [22] and correcting satellite imagery with multiple treatment and gap filling processes, retaining the original scales of the data. These data sources were paired with a geographic information system of all the residential areas and rice fields in the district collected via a participatory mapping project [23] to extract data from sentinel zones that represent where malaria transmission is likely occurring. Geostatistical models were trained using a Bayesian framework that allowed for spatio-temporal random effect structures to leverage information across community health catchments. Finally, the full workflow was integrated into a web application architecture that updates predictions continuously and provides information to local health actors in formats directly applicable to decision-making processes within the district (Fig. 1).

### Study area

Madagascar has seen an increase in malaria burdens since the beginning of global elimination efforts in the early 2000s [24], and is one of six countries in the WHO Africa region where malaria case incidence has increased by over 25% since 2015 [25]. The southeastern part of Madagascar experiences unimodal seasonal cycles of malaria [26], with overall higher prevalence rates than the central plateau regions [27–29]. Ifanadiana is a rural health district located in the Vatovavy region of southeastern Madagascar. The district’s population is approximately 200,000 people, the majority of whom live in rural, isolated villages over 1 h from a health centre [23]. The district is divided into 15 communes and 195 fokontany (the smallest administrative unit comprising one or several villages amounting to about 1000 individuals, which represents the community health catchment). Each commune contains at least one major primary health centre (PHC), and six of the larger communes contain a second, basic primary health centre that do not have medical doctors and provide more limited services (Fig. S5.1). Beginning in 2014, Ifanadiana has benefitted from a health system strengthening (HSS) intervention at all levels of the health system, from community health to the regional hospital, via a partnership between the Madagascar Ministry of Public Health (MMoPH) and the non-governmental organization Pivot [30].

Ifanadiana contains a variety of ecoregions, including a protected tropical rainforest in the west and warmer, humid zones near the eastern coast. There is an east–west elevational gradient from an altitude of 1400 m in the west to 100 m in the east. The dominant land covers are savanna and agricultural land for rice production. This diversity of ecoregions and climates translates into spatio-temporal heterogeneities in malaria burden in the district [4, 31].

### Data collection and treatment

#### Health data

The monthly number of malaria cases per community were collected from consultation registries at all primary health centers (PHCs) across the district, from January 2017-December 2020. Handwritten registries from each PHC were digitized, with each de-identified patient geolocated to the precision of a ‘*fokontany*’ (e.g. the catchment of one community health site). The number of malaria cases (both uncomplicated and severe), as confirmed by rapid diagnostic test (RDT), were aggregated by month for children under 5 years old, children aged 5–14, and adults aged 15 years and above. Because these data are collected at PHCs, they are passive surveillance data and contain primarily symptomatic cases. These raw data were adjusted for underascertainment due to spatial bias in healthcare access using the ZERO-G method [22], which accounts for whether the PHC benefitted from a long-term health system strengthening intervention, whether point-of-care user fees had been removed for the PHC, the number of staff at the PHC, the level of the services, and the distance from the PHC to the district office. The method also included additional identifiers of false-zeros based on the geographic coverage of the HSS intervention (see Supplemental Materials for more details). The ZERO-G method was applied separately to each age class due to age-specific care-seeking and symptomatic rates, using fokontany with annual consultation rates over 2 consultations/year as a reference. This translated the reported case numbers, which showed strong evidence of geographic bias, into estimated incidence rates of symptomatic malaria cases. The final data used in the model were the ZERO-G adjusted monthly case rates, aggregated across all ages, which represent reported cases assuming all fokontany had health-seeking rates equal to the reference fokontany (Fig. S5.2). While this does not account for asymptomatic cases as an active surveillance study would, it allows for the use of passive surveillance data by correcting for prominent data-quality issues.

Information on historical quantities of malaria diagnostics and treatment were used to validate model predictions and provide additional context for decision makers in the web application. These data were provided by the Madagascar Ministry of Public Health (MMoPH) at the monthly level for all major PHCs in the district beginning in 2017 and are updated continuously. This includes the number of febrile patients seen at the facility, the number of febrile patients tested via RDT, the number of malaria positive RDTs, and the number of RDT-positive patients treated with artemisinin-based combination therapy (ACT).

### Environmental indicators

**Fig. 2:**
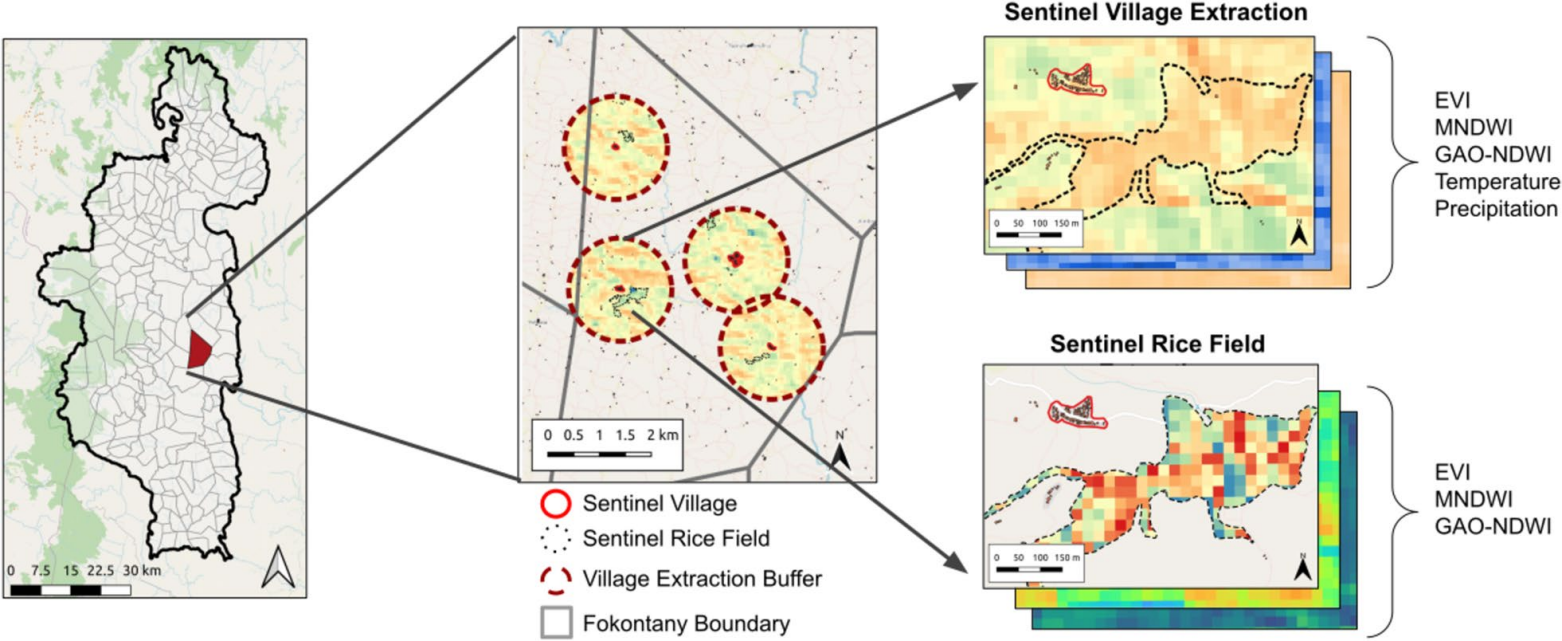
Process of extracting environmental indicators at a fine-scale from satellite imagery for sentinel villages and rice fields. Indicators at the sentinel village level are extracted using a 1 km buffer surrounding the village (red-dashed line), while indicators at the sentinel rice field level are extracted within the boundaries of the sentinel rice fields (black dashed line)

The surrounding environment can greatly influence the ecology of *Anopheles* mosquitoes, and, therefore, malaria dynamics themselves. A past study in Ifanadiana District identified variables related to forest and rice cover, elevation, and climate as potential drivers of malaria dynamics [4]. Data were therefore collected that described environmental dynamics, such as landcover, hydrology, and vegetation from the zones surrounding residential areas. Residential zones were located using a comprehensive dataset of the district of Ifanadiana collected via participatory mapping and available on OpenStreetMap [23]. This dataset includes over 25,000 km of roads, tracks, and footpaths, 20,000 rice fields, 5000 residential areas, and 100,000 buildings. The dataset used for this study was accessed on Nov 30 2021. Due to the size of this dataset and the processing time and resources it would require to extract data for all of these zones, sentinel sites of villages and rice fields were chosen to represent overall environmental conditions of a fokontany. Briefly, four sentinel villages of each fokontany were identified, determined by the number of buildings and field-based knowledge of communities. These sentinel zones contained on average 40% of buildings in a fokontany and 60% of all buildings within residential zones, with the remaining buildings usually standalone structures such as small houses or shelters used during the agriculture season, rather than residences. The analysis focused on the environment within 1 km of the centroid of these residential zones, assuming that mosquitoes’ flight distances were limited, following previous work in this region [32]. In addition, the largest rice fields adjacent to each sentinel village were identified, creating a dataset of potential larval habitat closest to human settlements. Sentinel rice fields are meant to represent the average rice field dynamics in a fokontany, and do not mean that those rice fields are necessarily an important source of mosquito populations. In instances where there were fewer than four villages or four rice fields near the sentinel villages in a fokontany, all of the available sentinel sites were used. This resulted in a total of 775 sentinel villages with a 1 km buffer and 769 sentinel rice fields that were used to extract environmental variables (Fig. 2). The representativeness of these sentinel sites were assessed and they were found to be very highly correlated (Spearman’s rho > 0.89 for all variables) with more complete spatial datasets (Supplemental Materials).

Landcover variables were calculated from a dataset derived from OpenStreetMap and Sentinel-2 satellite imagery, as described in Evans et al*.* [33] This dataset classified landcover into residential, rice field, savanna, forest, and open water at a 10 m resolution. The proportion of landcover that was a rice field within the 1 km buffer of the sentinel villages was estimated. This value was aggregated to the level of the fokontany by calculating a mean weighted by the population size of each sentinel village, multiplying the values by the proportion of the buildings in the residential area out of all buildings in all four sentinel residential areas (i.e. a building-weighted mean).

Two metrics representing spatial patterns in hydrology and standing water were derived from the SRTM 30-m digital elevation model (DEM). Because these metrics are derived from a static elevation model, they are constant over time. Topographic wetness indices (TWIs) estimate predicted water accumulation as a function of an area’s upstream catchment area and slope, and have been shown to predict malaria risk in regions with highly varied topologies [34, 35]. The TWI was estimated as $ln\left(\frac{a}{tan\left(\beta \right)}\right)$, where *a* is the surface catchment area of that pixel and β is the local surface topographic slope. The TWI represents, were it to rain, the combination of the amount of water expected to pass through each pixel following the topography of the area and the relative flatness of the pixels (with flatter pixels corresponding to areas where water is expected to pool or flow more slowly).

The spatial mean of the TWI at each sentinel village and rice field within a fokontany and the mean TWI at the village and rice field levels within a fokontany were calculated by taking a building-weighted and standard mean, respectively. From the same DEM, cells that served as sinks, or pixels with no outflow where water is likely to accumulate, were identified. The proportion of pixels that were sinks within a 1 km buffer of each sentinel village were extracted and aggregated to the fokontany-level value using a building-weighted mean. All topographic analyses were done using GRASS GIS [36] via the rgrass package in R [37].

Vegetation and water indices (EVI, MNDWI, GAO-NDWI) were derived from Sentinel-2 satellite imagery. These optical satellites have a revisit time of 5 days and a 10 m spatial resolution. The Sen2Chain Python tool [38] was used to download and process the Sentinel-2 imagery to level L2A before calculating three radiometric indices: the Enhanced Vegetation Index (EVI) [39], the Modified Normalized Difference Water Index (MNDWI) [40], and Gao’s Normalized Difference Water Index (GAO-NDWI) [41]. Each of these indices represents different aspects of rice field land cover relevant for *Anopheles* habitat. EVI is a function of the near-infrared (B8), red (B4) and blue (B2) bands of the Sentinel-2 imagery and represents vegetation health and vigor. MNDWI is a function of the green (B3) and shortwave infrared (B11) bands of the Sentinel-2 imagery and identifies areas of standing water. NDWI-GAO is a function of the near-infrared (B8) and shortwave infrared (B11) bands of the Sentinel-2 imagery and estimates moisture in the vegetation. Images with greater than 25% cloud cover were removed and indices were extracted at the sentinel villages and rice fields as described above (Fig. 2). Each sentinel zone (e.g. village or rice field) was less than 3 km^2^ and, therefore, very susceptible to measurement error and random fluctuations. To reduce this stochastic noise and interpolate measurements for dates with high cloud cover, the time series for each zone was smoothed by applying a cubic-spline to the series, using leave-one-out cross-validation to select the optimal number of degrees of freedom. This cubic-spline was then used to predict values on a weekly frequency for each index. Monthly means were calculated from these weekly values for each fokontany as follows: village-level indicators were aggregated by the building-weighted mean described above, and rice field indicators were estimated as the mean value of all sentinel rice fields in the fokontany. The final result was a monthly time series at the fokontany level for the three environmental indices at both the village and rice field sentinel zones.

Principal component analysis was used to create indices of rice field dynamics from EVI, MNDWI, and GAO-NDWI extracted from sentinel rice fields. First, the difference in each index at the rice field level from the index extracted at a buffer of 1 km surrounding sentinel village zones (Δvill-rice) was estimated to categorize dynamics specific to rice field environments. Second, the indice extracted at rice fields (EVI, MNDWI, and GAO-NDWI) was transformed into seasonal anomalies by standardizing each index within each calendar month (e.g. January, February, etc.) following Kaul et al*.* [42]. This seasonal anomaly represents how conditions differed in that year compared to the same calendar month over the long-term dataset. Third, the original three environmental indices extracted at the sentinel rice field zones were included. All three forms of the three indicators (Δvill-rice, seasonal anomalies, and original mean; 9 variables total) were used in the PCA, after having centred and scaled each one. The first three components, which contained over 74% of the overall variance, were selected to represent three indices of rice field dynamics (Tables S2.1, S2.2, S2.3). Rice Index 1 was strongly influenced by all three forms of the MNDWI indicator, and was positively associated with the amount of standing water in rice fields, representing wetter periods. Rice Index 2 was more strongly associated with EVI and NDWI-GAO and represented the vegetation dynamics of the rice fields, with higher values associated with greener vegetation in rice fields compared to the surrounding village. Rice Index 3 was most strongly associated with anomalies in the vegetation indices and represented anomalies in vegetation phenology (specifically increased vegetation) and the timing of the agricultural season (Table S2.3).

### Climate data

Meteorological stations are limited in Madagascar, with fewer than 30 stations across the country [43]. Satellite-derived climate data at resolutions less than 5 km are equally limited and, when present, suffer from cloud obstruction, particularly in the humid southeastern region. One solution is to aggregate data to a coarser resolution or over multiple time periods, but this results in spatial scales too coarse to accurately represent hyper-local conditions. This lack of fine-scale, accurate data was considered in the choice of data sources and processing of both temperature and precipitation data. Land surface temperature (LST) was estimated via a gap-filling algorithm that combines climatology from fine-scale MODIS imagery with modelled surface temperature from the Climate Forecast System Version 2 (CFSv2) to create daily estimates of LST at a 1 km resolution [44] This method has been validated globally and performs well in this region of Madagascar, with a Root Mean Square Error of less than 2 °C. The analysis specifically used the MODIS Aqua Daily Land Surface Temperature, which was averaged to a monthly value after gap-filling. This represents an improvement in resolution over the 8 km resolution corrected data available directly via MODIS at the monthly level. Precipitation data was obtained from the NOAA Africa Rainfall Climatology v2 (ARC2) daily precipitation dataset via the rnoaa package in R [45]. This dataset was selected for its high reported accuracy for this region of Madagascar [43]. The daily precipitation was summed by month to obtain the total monthly precipitation for each 0.1 × 0.1 degree (~ 10 km) pixel. Both climate variables were extracted using a sentinel village 1 km buffer and aggregated to the fokontany-level via a building-weighted mean, as described above.

### Socio-demographic data

Socio-demographic data were collected from 2014–2021 via the IHOPE cohort, a longitudinal survey based on the Demographic and Health Surveys, conducted in about 1600 households of Ifanadiana district distributed across 80 spatial clusters [46]. Briefly, a two-stage sampling design was used to sample 40 clusters at random within each of two strata, the initial HSS intervention catchment and the rest of the district. Twenty households per cluster were then randomly selected to be surveyed. Further details on the IHOPE longitudinal survey can be found in Miller et al*.* [46, 47]. The IHOPE cohort collects information on household-level socio-demographic, health, and socio-economic indicators. Household wealth scores, calculated following standard DHS methods, have historically been stable over time, and are not expected to shift drastically between survey years [48]. Data on household wealth scores were extracted to the fokontany level following Evans et al*.* [48].

In addition to household-level demographic data, the model included variables representing the geography of each fokontany, specifically the distribution of houses and the distance to primary health centers. Residential areas are clustered within Ifanadiana district and a fokontany-wide estimate of building density does not accurately represent the population density experienced by an individual. Therefore, a relative building density was calculated by estimating the density of buildings within 100 m of a building for all buildings within each fokontany. The median of this value was used to obtain fokontany-level estimates. The distance to a primary health centre was estimated for each building over the full transport network using the Open Source Routing Machine (OSRM) routing algorithm via the osrm package in R [49], and the average distance was calculated for each fokontany.

#### Health intervention data

The study period intersected with a time of on-going health system interventions known to impact malaria dynamics. Over the past decade, Madagascar has conducted a mass long-lasting insecticide-treated bed net (LLIN) distribution every three years beginning in October 2015. The effect of bed nets decreases over time due to waning bioefficacy and functional integrity [50–52]. Therefore, a variable was included in the model to represent this waning over time by calculating the number of months since the most recent bed net distribution. This variable is referred to as the months since LLIN distribution. From October 2019 thru December 2021, a pilot proactive community care intervention was implemented in one commune in the district, increasing treatment rates of malaria by nearly 40% [53]. This heterogeneity in treatment rates caused by interventions was accounted for by including a binary variable for months and fokontany when the proactive care intervention was in place. Finally, although the data were already adjusted for geographic bias in health-seeking behaviours via ZERO-G, an artifact of this bias remained. The distance to the nearest PHC was therefore included in the model, allowing for a non-linear relationship via a penalized smoothing spline.

#### Statistical model

The analysis implemented a Bayesian spatio-temporal model via an Integrated Nested Laplace Approximation (INLA) model that included hierarchical random effects [54]. Bayesian hierarchical models are particularly useful for analysing spatio-temporal data because of their ability to leverage random effects across space and time to account for spatio-temporal correlation and more accurately estimate random effect coefficients when data are of low quality or missing. The spatio-temporal covariance in the data was accounted for in two ways. First, a cyclical temporal term by month of the year was included via a first order random walk, estimated for each commune. Second, spatial covariance by fokontany was included via the Besag, York, and Mollie spatial model [55, 56], which includes an unstructured random effect for each fokontany in addition to a Besag model for the spatial structure. Predictor variables were inspected for normality and log-transformed when necessary, then scaled and centered to aid with model convergence.

Dynamic variables were lagged by 3 months to account for delays in the effect of environmental and climatic variables on malaria transmission and to allow for predicting malaria trends into the future. A supplementary analysis explored including non-linear effects of predictor variables via penalized splines, but found that it performed similarly to a model including only linear effects while requiring a significantly longer computation time (details reported in the Supplementary Materials). The more parsimonious linear model is presented here. This resulted in a total of fourteen covariates in the model, six of which are lagged, dynamic variables updated monthly (Fig. S5.3, Table 1). The INLA model was fit to ZERO-G adjusted, monthly case rates at the fokontany level via a zero-inflated negative binomial distribution using a log-link. It was trained on data from January 2017 thru December 2020 and used to predict future disease incidence for the MEWS.

**Table 1:** Predictor variables used in the INLA model to forecast malaria incidence

Variable	Temporal Frequency	Spatial Resolution	Temporal Lag	Source
Building Density	Static	Fokontany	NA	OpenStreetMap
Months since LLIN Distribution	Monthly	Fokontany	0 months	MMoPH
Proactive CHW	Monthly	Fokontany	0 months	Pivot
Proportion Ricefield	Static	10 m (Village Extraction)	NA	Pivot
Proportion Sink	Static	30 m (Village Extraction)	NA	SRTM
Topographic Wetness Index (Ricefield)	Static	30 m (Village Extraction)	NA	SRTM
Topographic Wetness Index (Village)	Static	30 m (Ricefield Extraction)	NA	SRTM
Wealth	Static	Fokontany	NA	Pivot
Enhanced Vegetation Index (Village)	Monthly	10 m (Village Extraction)	3 months	Sentinel-2
McFeeter’s Normalized Difference Water Index (Village)	Monthly	10 m (Village Extraction)	3 months	Sentinel-2
Precipitation	Monthly	0.1 degree (Village Extraction)	3 months	ARC2
Rice Index 1	Monthly	10 m (Ricefield Extraction)	3 months	Sentinel-2
Rice Index 2	Monthly	10 m (Ricefield Extraction)	3 months	Sentinel-2
Rice Index 3	Monthly	10 m (Ricefield Extraction)	3 months	Sentinel-2
Land Surface Temperature	Monthly	1 km (Village Extraction)	3 months	MODIS

Out-of-sample prediction tests were performed across both space and time to assess the model’s predictive capabilities via leave-one-out cross-validation. Spatial out-of-sample assessment was done by commune, leaving one commune out of model training and predicting incidence in that commune, resulting in 15 separate tests. Temporal out-of-sample assessment was done by year, where each sample omitted from model training corresponded to a year of data from 2017 to 2020. The model’s predictive ability was assessed via the IQR of the absolute error, which is more robust to outliers than the Root Mean Square Error (RMSE), and the Spearman’s correlation coefficient (ρ) between the predicted and estimated incidence rates.

A motivation for the creation of this dashboard was the high frequency of disruptions to diagnostic and medical stocks (“stock-outs”) observed in malaria-endemic regions of Madagascar, and the need for better guidance to plan future stock use. Therefore, a rudimentary, complimentary exercise was conducted to demonstrate how malaria forecasts could be used to inform decisions surrounding medical commodities. This exercise retroactively compared malaria treatment needs (i.e., ACT) estimated by the model to those estimated following standard stock ordering protocol. The standard MMoPH protocol uses the following formula to order four months of stock every two months (e.g. the two-month order quantity), with the four month estimate serving as a buffer [57]:

$$Expected\ stock\ use=\frac{\left(Total\ stock\ use\ during\ prior\ three\ months \times 30\right)}{90-\left(days\ with\ stock\ out\ or\ facility\ closure\right)}\times 4$$

A key number in the commodity ordering process is the value used to represent the average monthly cases, which is then multiplied by four. Two ways of calculating this value were compared: 1) using the MMoPH formula based on the total use during the prior three months and 2) using the SMALLER MEWS to estimate the average monthly cases over the next two months.

The expected ACT use based on the MMoPH formula and the SMALLER model for each two month period were calculated and compared to actual ACT use from January 2017–December 2020. To do this, the number of cases per fokontany expected to seek care at a PHC were back-calculated by rescaling the total number of cases per fokontany. This assumed a binomial probability of observation of each case at a PHC equal to the sampling intensity estimated via the ZERO-G method. The cases that did not seek care were assumed to remain at the community health catchment, where they may seek care at the community health site, be treated via an advanced strategy, or remain untreated. The proportion of cases attending each PHC from each fokontany were then predicted based on the historical distribution of consultations from each fokontany to each PHC from 2018–2019. This subset of the data was chosen in order to use the most recent and most complete consultation data that did not suffer from bias due to the COVID-19 pandemic which began in 2020. These numbers were then aggregated to the level of the PHC, resulting in the number of malaria cases predicted to seek care at each PHC during each month, or the predicted monthly ACT need. The average of these two months were multiplied by four, following the equation above, to calculate the SMALLER two-month ACT order quantity. To calculate the estimated ACT order quantity via the MMoPH protocol, the equation above based on the stock use during the prior three months was followed. The two-month order quantities calculated via the SMALLER MEWS were compared to those estimated via the MMoPH protocol, and both were compared to the historical ACT requirements for 15 major PHCs from January 2017–December 2020. The historical ACT requirements corresponded to the number of positive RDT tests seen by the PHC over that two month period (e.g. positive cases in need of treatment). The relative performance of the SMALLER and MmoPH estimations were assessed by estimating the median absolute error and under-estimation rate of the two estimations when compared to the historical ACT requirement.

### Automating the workflow for a MEWS web application

The remote sensing and statistical model workflow for a MEWS web application were automated via the targets package [58], which creates a Make-like pipeline for R scripts. The use of a Make-like workflow is especially beneficial for deploying a MEWS in a resource-limited setting because only those data sources and tasks that are not up to date are rerun in the monthly update, conserving computational and network resources. The targets pipeline “backend” is linked to an R shiny “frontend”, which contains the web application user interface (Fig. 1). The workflow updates monthly, collecting new environmental variables and creating updated forecasts that are then available online for use by local health actors.

The targets pipeline contains the Extract-Transform-Load and Analysis modules of the application (Fig. 1). Static data are loaded into the project one time, pre-processed and formatted for the model. Dynamic data are then updated monthly on the tenth day of the month, to allow for latency in data collection, before being combined with the static data to use in prediction. A combination of tools were used to collect this data semi-automatically. Google Earth Engine scripts, which process temperature data, are manually run via the Online Code Platform, and processed data are added to the project, where they are tracked via the targets workflow. Sentinel-2 indices are updated monthly via the Sen2Chain tool implemented on servers hosted at SEAS-OI Station at Université de la Réunion, where the data are then provided through HTTP. The data source url is added to the targets workflow, which then observes the HTTP file for changes. If a change is made to the file, the new data are automatically included in the next update. Because the targets workflow tracks the downstream flow of data, when the raw index data are updated, all downstream features derived from this, such as the rice indices, are also updated. The workflow is semi-manual, in that the automatic pipeline is run under supervision, so that the input data and resulting predictions can be validated by a subject expert before being made publicly available. This is done via a quarto document that automatically renders after the workflow is completed, providing information on the environmental input data and output predictions.

## Results

### Malaria case data

**Fig. 3:**
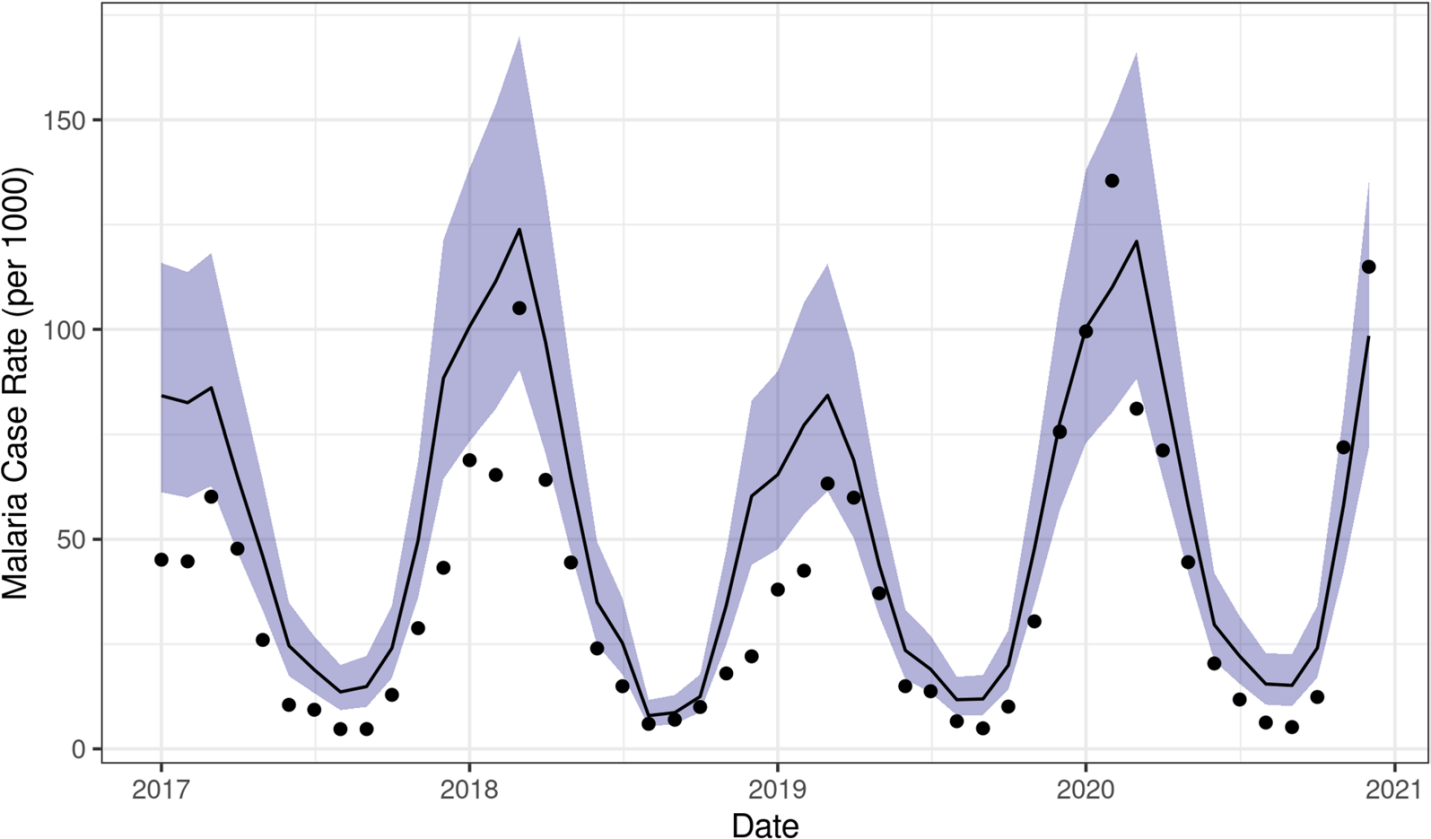
Comparison of SMALLER predicted incidence rate (line) and estimated case rate (points) per 1000 population at the District level. Shaded area represents 95% CI

In total, there were 107,739 reported malaria cases in Ifanadiana district from January 2017–December 2020. Missingness of data (*i.e.*, registers not available) was 2.21%, or 207 of 9360 total month by fokontany samples. The median annual fokontany-level reported case rate was 68.8 cases per 1000 individuals (95% CI: 0 – 445). Variance was high in the initial dataset, with a standard deviation of 17.1 cases per 1000 per month, nearly twice the mean of 9.6 cases. After applying the ZERO-G method, which adjusts for underascertainment due to spatial bias in healthcare access, the estimated total number of symptomatic malaria cases was 377,211, with a median annual estimated case rate per fokontany of 357 cases per 1000 individuals (95% CI: 4.40–1621). The highest estimated case rate was observed between the months of November – April (i.e. the warmer, rainy season), ranging from 37.2 – 77.1 cases per 1000 individuals per month (Fig. 3, Fig. S5.6). The median of the average annual estimated case rate from 2017–2020 was 1626 cases per 1000 in the 25% of fokontany experiencing the highest rates and 272 cases per 1000 in the 25% of fokontany experiencing the lowest rates.

### Model performance and results

The INLA model was able to accurately reproduce community-level malaria incidence rates based on ZERO-G estimated case rates (Fig. 3). When applied to the full dataset, it achieved a median absolute error of 16.99 cases per 1000 individuals per month (IQR = 7.30 – 38.93), equivalent to less than 1% of the variance of the estimated rates. The predicted and estimated case rates were significantly positively correlated (Spearman’s p = 0.647, p-value < 0.0001, Fig. 3, Fig. S5.6). Similarly, the model performed well when assessed via cross-validation across space and time (Table 2). Predictive ability was similar across communes, with the exception of one commune in the south of the district (Fig. S5.4), where accuracy was much lower than in the other 14 communes. The model performed well at predicting incidence in out-of-sample years. In particular, the full model performed better on out-of-sample data in 2020 than on in-sample data, evidence of the models’ ability to forecast forward in time (Fig. S5.5).

**Table 2:** Performance metrics of the predictive model when assessed via leave-one-out cross-validation across time and space

	Temporal cross-validation (by year)		Spatial cross-validation (by commune)	
	Null Model	Full Model	Null Model	Full Model
In-Sample MAE	36.23 (35.64–36.5)	15.93 (15.46–16.69)	36.24 (35.22–37.82)	15.93 (15.35–17.03)
Out-of-Sample MAE	36.26 (35.6–38.13)	15.91 (14.03–17.25)	40.39 (22.75–67.25)	18.55 (9.53–48.72)
In-Sample Spearman’s ρ	0.10 (0.09–0.12)	0.66 (0.63–0.69)	0.10 (0.07–0.13)	0.66 (0.65–0.68)
Out-of-Sample Spearman’s ρ	0.10 (0.05–0.13)	0.66 (0.54–0.74)	0.05 (− 0.12–0.27)	0.67 (0.41–0.84)

The null model corresponds to a model that only estimates a district level mean without any covariates. Values are the mean performance metrics with the range of metrics in parentheses. Median absolute error (MAE) represents the median absolute difference between model predictions and the true values, with lower values representing better model performance. Spearman’s ρ represents the correlation between the model predictions and the true values, with values closer to one representing better model performance

**Fig. 4:**
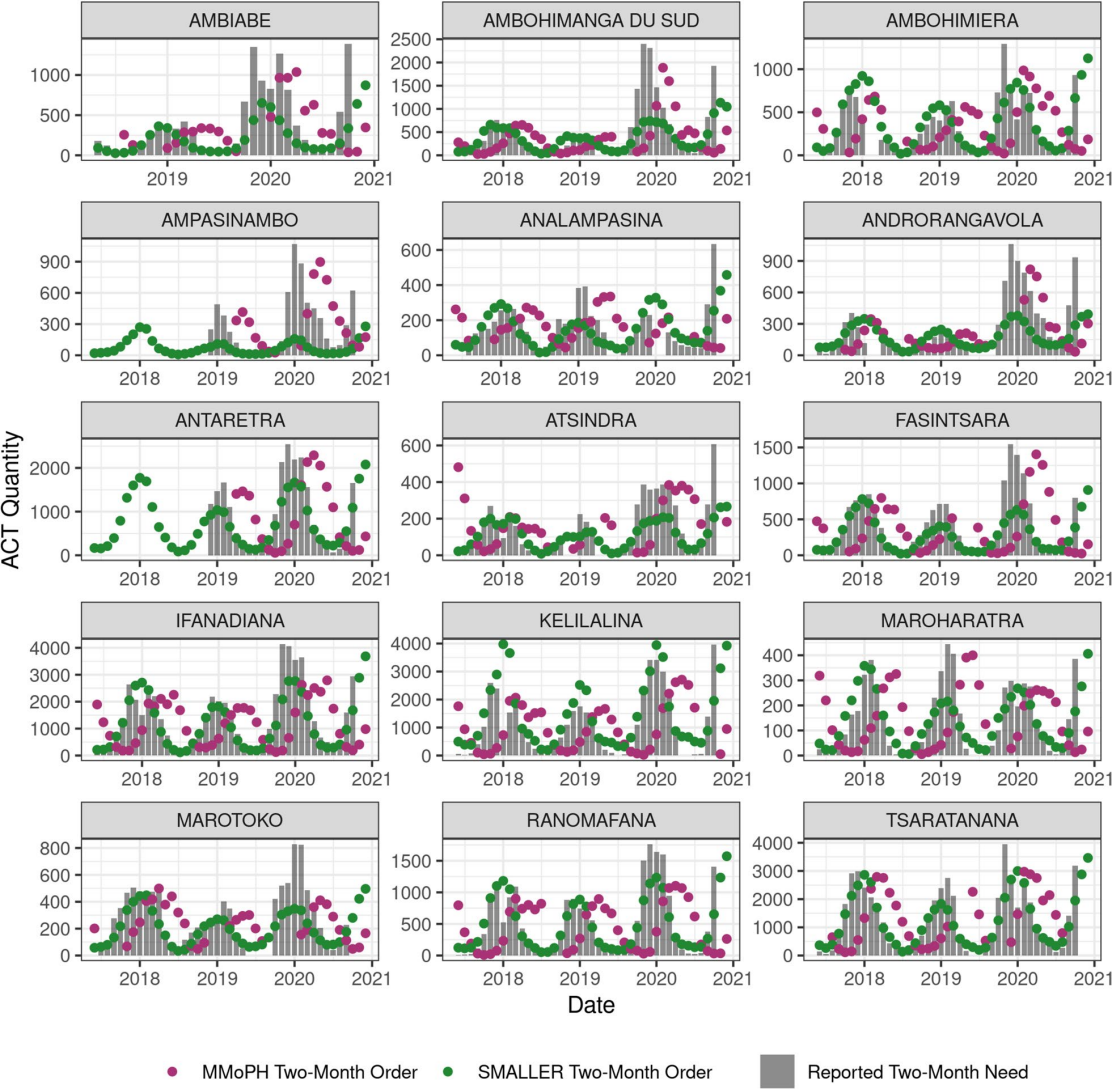
Two-month ACT order quantities based on the SMALLER MEWS more closely matched reported two-month use than order quantities based on the MMoPH calculation. Reported historical ACT use is compared with hypothetical ACT order quantities based on two calculation methods for the 15 advanced PHCs in Ifanadiana District

Commodity quantification using the SMALLER MEWS were compared with existing MMoPH processes. This involved estimating the two-month order quantity of ACTS based on the predicted malaria incidence (SMALLER method) or the ACT use over the prior three months (MMoPH method). Both methods were then compared to true historical ACT needs. Reporting of historical stock requirements was incomplete, with 13 of 15 PHCs missing the number of positive RDTs during at least one month. However, only 2 PHCs were missing more than 5 months of data over the 48 month period. All comparisons between the MMoPH and SMALLER method were only done for complete pairwise sets. The reported historical ACT requirement ranged from 15,624 to 38,154 annually across the whole district, increasing overtime (Fig. 4). The reported average two-month ACT requirement for a PHC was 292 (median: 138, range: 2–2066). Following the MMoPH method, the average two-month order quantity of ACTs was 549 (median: 290, range: 6–2973). Following the SMALLER method, the average two-month order quantity of ACT was 508 (median: 240 range: 6–3974). The median absolute error between the calculated two-month order quantity and the reported two-month need was 113 for the SMALLER method and 286 for the MMoPH method, an improvement of over 60%. While the average two-month order quantity was similar, the seasonality of two-month order quantities varied greatly between the two methods, with the SMALLER method better tracking the seasonality of the reported two-month ACT use (Fig. 4). In addition, the SMALLER method under-estimated the ACT need 14.2% of the time, compared to 33.8% of the time with the MMoPH method.

**Fig. 5:**
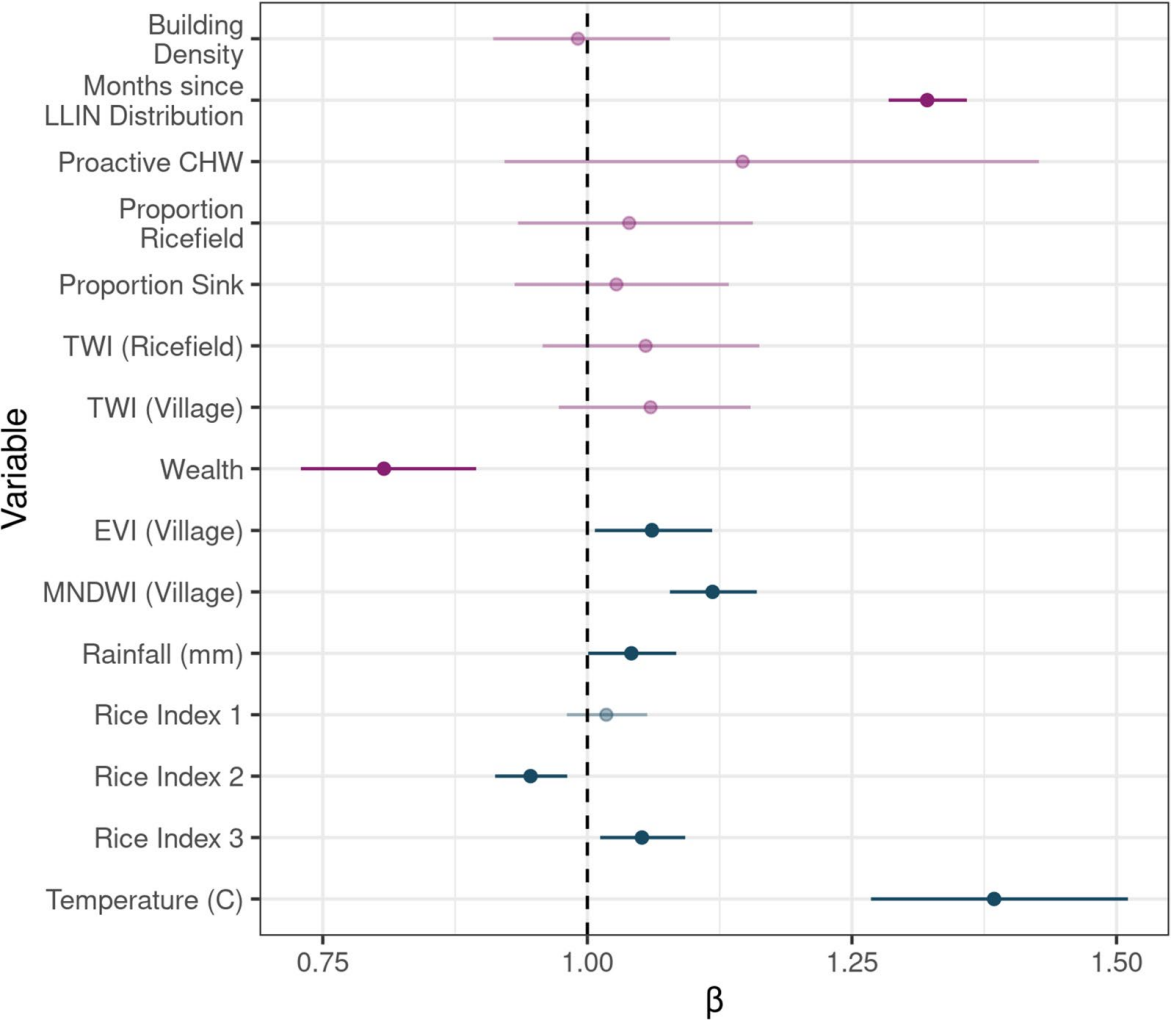
Model coefficients of the primary INLA model. Static variables are colored in purple and dynamic variables, which are lagged by 3 months and updated monthly, are colored in blue. Points represent the median value of the coefficient and error bars the 95% confidence intervals (CI). Those points whose CI overlaps 1, and therefore do not exhibit a statistically significant relationship with malaria incidence, are represented in faded colors. TWI: Topographic Wetness Index (a measure of likelihood of standing water based on topography), EVI: Enhanced Vegetation Index, MNDWI: McFeeter’s Normalized Difference Water Index (a measure of standing water). The three Rice Indices are a composite measure of EVI, MNDWI, and NDWI-GAO (a measure of humid vegetation)

While the objective of this model was to predict future malaria cases based on established relationships with environmental variables, and not draw inference about these relationships, this does not preclude the exploration of these relationships established via the statistical model. The time since the previous LLIN distribution was strongly associated with higher estimated incidence rates of malaria (Fig. 5, Table S6.1). Fokontany with higher socio-economic levels tended to have a lower estimated incidence rate of malaria (Fig. 5, Table S6.1). The majority of the 3-month lagged dynamic environmental variables were significantly associated with estimated malaria incidence (Fig. 5, Table S6.1). Malaria incidence was strongly associated with temperature and MNDWI at the village-level (Table S6.1, Fig. 5). It was also associated with EVI at the village-level, rainfall, and Rice Indices 2 (vegetation dynamics) and 3 (seasonal anomalies), although these coefficients were smaller than for temperature and MNDWI (Table S6.1, Fig. 5).

### SMALLER MEWS web application

**Fig. 6:**
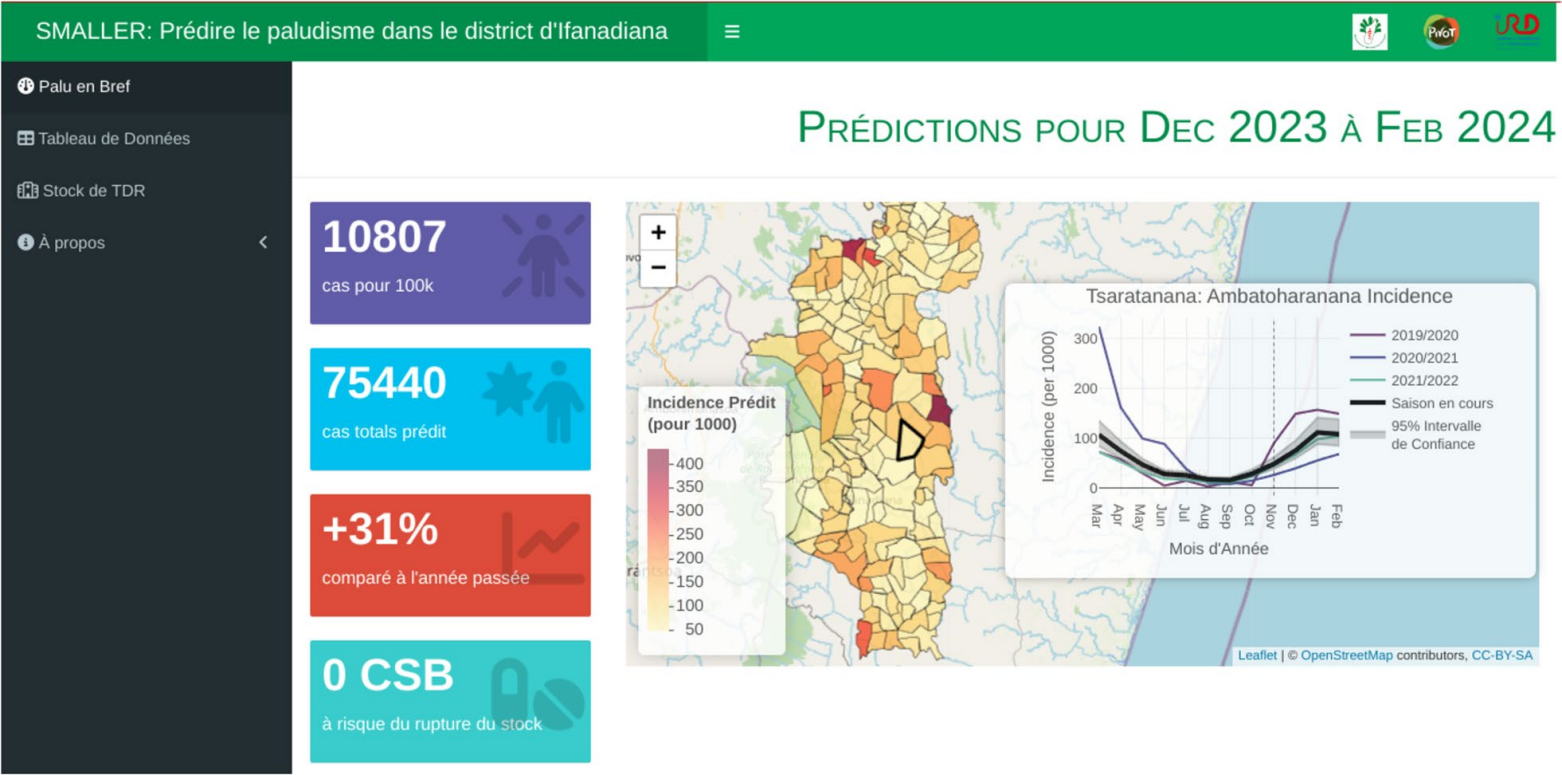
Screenshot of the landing page of the SMALLER MEWS web application. The side panel serves to navigate between the pages of the site, including a page to download the data and explore predicted needs at the community and primary health centre level. The web application can be accessed at https://smaller.pivot-dashboard.org/

The INLA model was integrated within the automated workflow to predict malaria incidence three months in advance for each fokontany. These predictions are accessible via a dashboard-style web application which provides hyper-local information relevant to programme managers and personnel working across the health system in Ifanadiana district. The landing page of the application provides an overview of malaria burden across the district over the next three months (Fig. 6). This includes alert buttons displaying four key indicators for district health personnel: the total number of cases, the total incidence, the malaria burden compared to the prior year, and the number of health clinics expected to receive more cases than the prior year. Also on this landing page is a map of the incidence in the district, displayed at the spatial scale of the community health catchment. Selecting a fokontany on this map opens a window which displays a time series of the forecast malaria incidence, with historical time series included for context. These data can be explored further via a table that displays these values by month and fokontany. The table can be subset interactively and all of the data are available for download as a csv file.

One objective of this dashboard was to address health actors’ concerns regarding disruptions to diagnostic and medical stocks. Modules help inform the risk of stock disruptions for each major PHC and the expected number of cases requiring treatment at the community health catchment. The module displaying stockout risk for each PHC compares the predicted number of cases expected to seek care at a PHC with the historical ACT use over the past two years. The number of malaria cases not captured by PHCs and remaining at the community health level were estimated, presented in a separate module. These cases could seek care at a community health site, be identified during proactive community health visits, or remain untreated. These functionalities are meant to provide granular information for district managers and health workers for context-specific decision-making.

## Discussion

Advances in disease analytics and forecasting, coupled with the increased availability of timely health data and fine resolution remotely-sensed satellite information, promise a new era of precision public health which will allow the delivery of “the right intervention to the right population at the right time” [59]. Yet, there is currently an important gap between the spatio-temporal scales at which these tools are available, and the much finer scales necessary to inform local programme implementation for improving disease surveillance and control. This study developed a hyper-local malaria early warning system (MEWS), SMALLER, for use by a health system strengthening programme serving a rural health district in southeastern Madagascar. SMALLER combines fine-resolution information on static hydrological and socio-demographics with fine-resolution dynamic data derived from satellite imagery and climate models that are updated on a monthly frequency. SMALLER forecasts malaria incidence at the community scale (a village or group of villages) up to three months in advance, improving correlations with out-of-sample data by nearly 500% compared to a null model. When compared to existing methods for calculating ACT order quantities, it reduced the frequency of underestimating the true need by more than half, demonstrating the potential utility of the SMALLER MEWS for decision making. Integrated into a web application, SMALLER provides real-time access for local health actors to the MEWS predictions to aid in context-specific decision-making.

SMALLER contributes to a rapidly growing ecosystem of disease forecasting tools developed to aid in decision making. A recent review found that of the 37 existing tools used for the modelling of climate-sensitive infectious diseases, 16 of them focused exclusively on malaria [60]. The sensitivity of malaria to climate and environmental variables makes it an ideal candidate for disease forecasting efforts [6]. Indeed, this study found that ecological variables related to temperature and vegetation dynamics were strongly associated with malaria incidence, in agreement with past work [4, 32, 61, 62]. Interestingly, after accounting for seasonal dynamics via a cyclical temporal structure, MNDWI was more strongly associated with malaria incidence than precipitation. This variable was collected at a finer spatial resolution (10 m vs. 10 km) that represented local standing water dynamics. Weather stations are rare across the African continent, resulting in downscaled gridded precipitation data of low-quality [63]. This suggests that indices derived from satellite imagery may be more useful than those from coarser precipitation models for malaria prediction at local scales in areas with poor weather station coverage.

A feature unique to the SMALLER MEWS is its ability to integrate disease forecasts with information on historical stock quantities and disruptions. The ability of stock-outs to hinder progress towards malaria elimination has been highlighted since the introduction of ACTs nearly fifteen years ago [64, 65]. A stock-out not only prevents an individual patient’s treatment, but can increase healthcare costs when patients must seek treatment at private facilities [66]. Lower or delayed treatment rates can, in turn, allow for increased severity, onward transmission, and higher population-level prevalence rates [67]. A multinational study of eight sub-Saharan African countries found that, in contrast to the other seven countries in the region, Madagascar experienced a decrease in malaria diagnostic availability in public health facilities from 2010 to 2015 [68]. Both RDTs and ACTs are delivered to public health facilities via a “pull system”, where health facility managers manually fill in quarterly orders for medicines and supplies, which are delivered from the capital to district depots and eventually individual public facilities’ pharmacies. Per national policy, the quantity requested is a function of the amount of materials dispensed during the prior quarter. Given the strong seasonality of malaria in Ifanadiana, particularly its exponential growth between the months of October–January, basing future needs on recent use can result in both under- and over-estimation of the quantities needed, depending on the season (Fig. 4). Although the SMALLER MEWS offered a significant improvement in performance over existing methods of calculating two-month stock orders, it continued to overpredict stock needs with a median error rate of over 100 doses. While the objective of the SMALLER MEWS is to predict incidence rates at the fokontany, this limitation does highlight the difficulty in transforming and aggregating incidence rates at the community level into expected intake rates at PHCs. Health-seeking behaviours and stock requirements are determined by complex economic and behavioural processes that govern the health system, and therefore fluctuate greatly from month to month. Particularly at the hyper-local scale, relatively small stochastic events, such as an impromptu active surveillance campaign undertaken by a PHC manager or a several-day disruption of a transportation route, can drastically influence the number of cases seen at the health centre that month. SMALLER MEWS was unable to capture these months with uncharacteristically high or low requirements for the season, particularly in more recent years during the COVID-19 pandemic, but predictions could be improved through the integration of more recent health system data to inform the back-calculation or by modelling reported case rates directly.

Limitations to the scaling-up of SMALLER are primarily related to data constraints. An ideal MEWS would be directly connected to an electronic HMIS, such as DHIS2 in the case of Madagascar and many other countries, to facilitate the timely incorporation of the most recent disease and stock data. However, HMIS data are often reported at the scale of the PHC catchment, which comprises dozens of villages across hundreds of square kilometres, and rarely at spatial scales relevant to local targeting by community health workers and mobile teams. Handwritten PHC registries were manually digitized to obtain the granular dataset needed to train the statistical model in the SMALLER MEWS, a resource- and time-intensive process that is not scalable at a national level for routine surveillance. In addition, this resulted in a relatively short time series on which to train the statistical model. However, the expansion of eHMIS systems and mobile technology such as commCare or DHIS2 tracker, which include information on patient residences, will make the integration of HMIS data into a national, highly granular MEWS more feasible in the future. Indeed, an electronic data collection programme at PHCs was established in the district of Ifanadiana in October 2023, and will be integrated into a MEWS in the coming years as it expands to cover the entire district. Environmental data can also be limiting, not necessarily due to their availability but rather to the computational and technical resources required to access and process datasets at local scales [6]. For example, a day of Sentinel-2 imagery for the country of Madagascar contains over 60 images, each 500–700 MB in size. Downloading this volume of data would be difficult with limited internet connectivity and the treatment and processing of the images would require a high-processing computer, given their quantity and size. Services which process satellite imagery on a remote server, such as Google Earth Engine, AWS, or MOSAIKS [69] provide access to processed data without downloading the images themselves, but still require paid accounts and geospatial expertise to use. The on-going push for building disease forecasting capacity among public health actors and organizations will require further investment to make these data available in regions with low connectivity and computational resources for a successful and timely integration into health information systems.

The SMALLER MEWS demonstrates the feasibility of predicting malaria incidence rates at the community-level, using fine-scale environmental data, and this study offers an example of how this data could be used be health actors, specifically heads of PHC who oversee primary care and community programmes. However, the implementation of such a MEWS in the field would necessarily require additional policy changes and support in order to be successful. First, lack of internet connectivity could limit access to the MEWS, particularly in remote areas. Second, local health actors may not have the agency nor the budget to make programmatic decisions in response to this data [70]. In Madagascar, community programmes are planned and managed by the PHC and District teams, with CHWs serving primarily as implementers. While the District office has some programmatic freedom, they are necessarily limited by budgets and policies defined at the regional or national level. For this reason, the implementation of a MEWS should involve actors at multiple levels of the health system and the tool itself should be accredited by the Ministry of Health and integrated with existing health and logistics management information systems. A participatory approach, which involves multiple health actors in the co-creation of the model and decision support tool, offers one way to surmount many of these obstacles [71, 72]. Indeed, an in-progress continuation of the SMALLER MEWS project uses a participatory approach to co-create an EWS for multiple diseases with MMoPH partners that will ultimately be integrated into an application for DHIS2, the HMIS used by the MMoPH.

## Conclusions

While recent advancements in data availability and statistical modelling have led to rapid growth in the development of MEWSs, few have been developed at the community scale required by certain public health interventions. This study combines fine-resolution routine health data and environmental data from satellite imagery into a MEWS capable of generating predictions at a hyper-local scale. Cross-validation exercises revealed that the statistical model on which the MEWS was based had high predictive capacity across both space and time when applied to out-of-sample datasets. An additional exercise demonstrated how this information could be used to inform one example of decision making: quantifying ACT needs. While this relatively simple example offered an improvement over existing methods, it over- and under-predicted ACT needs by over 100 doses on average. This highlights a limitation of both the SMALLER MEWS and MEWSs in general regarding their ability to adequately account for the complex processes determining medical stock requirements and their integration with stock management systems. Future work should focus on how to integrate the already highly-predictive environmentally-driven MEWS into more complex models of health system functioning and existing HMIS architectures to better understand and predict resource needs.

## Supplementary Information

Supplementary Material 1.

## Data Availability

Data and code needed to reproduce these analyses can be found in a figshare repository (doi:10.6084/m9.figshare.25516318). The web application’s source code can be found at https://gitlab.com/pivot-sci-apps/smaller-backend and https://gitlab.com/pivot-sci-apps/smaller-shiny.
