# Supplementary material for: Increasing the resolution of malaria early warning systems for use by local health actors
Source: Malar J. 2025 Jan 30;24:30. doi: 10.1186/s12936-025-05266-0 (PMC11780933; doi:10.1186/s12936-025-05266-0)
Supplement: Supplementary file 1 — Supplementary Material 1. [file 12936_2025_5266_MOESM1_ESM.docx]

**SUPPLEMENTAL MATERIALS**

**Increasing the resolution of malaria early warning systems for use by local health actors**

Michelle V Evans, Felana A Ihantamalala, Mauricianot Randriamihaja, Vincent Herbreteau, Christophe Révillion, Thibault Catry, Eric Delaitre, Matthew H Bonds, Benjamin Roche, Ezra Mitsinjoniala, Fiainamirindra A Ralaivavikoa, Bénédicte Razafinjato, Oméga Raobela, Andres Garchitorena

Table of Contents

1. Investigation of Sentinel Sites
2. Creating Rice Indices
3. Configuring the ZERO-G adjustment for the study zone
4. Comparing Non-linear vs. Linear Model Performance
5. Supplemental Figures
6. Supplemental Tables
7. French Abstract
8. References

# S1. Investigating Sentinel Sites

Because the objective of this study was to model malaria incidence at fine-spatial scales, we aimed to use the finest resolution satellite imagery available for the region, specifically 10m resolution imagery from the Sentinel-2 satellite. A disadvantage of using 10m resolution imagery is that its processing and extraction can be extremely resource intensive, a burden that scales with the number of zones from which data is extracted. For this reason, we chose to use sentinel sites to represent the environment within the fokontany. We used two sets of sentinel sites: 1) sentinel villages, corresponding to the 4 largest villages per fokontany and 2) sentinel rice fields, corresponding to the largest rice field adjacent to each sentinel village. To extract data for these sentinel sites, we applied a 1km radius buffer around the centroid of the sentinel village and used the border of the sentinel rice field. Because not all fokontany had more than four villages and some sentinel villages did not have adjacent rice fields, this resulted in a total of 775 sentinel villages and 769 sentinel rice fields.

The cartography and census teams for Ifanadiana estimate that, on average, only 40% of buildings in a fokontany are active households, with the rest representing storage, business, or temporary residences used during the harvest season, particularly those located further from larger clusters of buildings (Randriamihaja et al. 2024). Villages are not randomly dispersed throughout the district, but are clustered around larger population centers, with most in close proximity to the sentinel villages: 79.1% of the total area of residential zones in the district and 68.4% of all buildings are contained in the 1km buffer surrounding the sentinel villages. The choice of 1 km buffer was driven by the average flight of *Anopheles* mosquitoes and past work in the region. However, the 1km buffer also ensured that all of the buildings in a village were included within the buffer. The widest sentinel village was 0.49 km, with 50% of sentinel villages much smaller (<0.35 km). The 1km buffer therefore encompassed all residences in a village, plus the nearby surrounding area.

We further validated these sentinel sites by comparing the extracted environmental variables for this subset of sites to an expanded selection of villages and rice fields. The expanded set included the ten largest villages per fokontany and the twenty largest rice fields adjacent to this set of villages. As above, not all fokontany have ten villages or twenty rice fields. In this case, we choose all residential villages or all rice fields within the fokontany. This resulted in 1224 sentinel villages and 2844 sentinel rice fields. We then calculated fokontany-level means by taking the building-weighted mean of sentinel villages or the mean of rice fields within the fokontany and compared the original and expanded sentinel datasets. The image processing and extraction process took 54 hours for this expanded dataset.

The two subsets were highly correlated for all variables, with Spearman’s rho ranging from 0.899 – 1 and scatterplots revealing clear linear relationships (Fig. S1.1.). Dynamic variables extracted at sentinel rice fields (e.g. EVI. MNDWI, NDWIGAO) had the most variation due to the smaller size of the extraction zones compared to village extraction zones, which rendered them more sensitive to random noise. Overall, however, the original, smaller subset of sentinel sites seemed to accurately represent the larger subset.

#
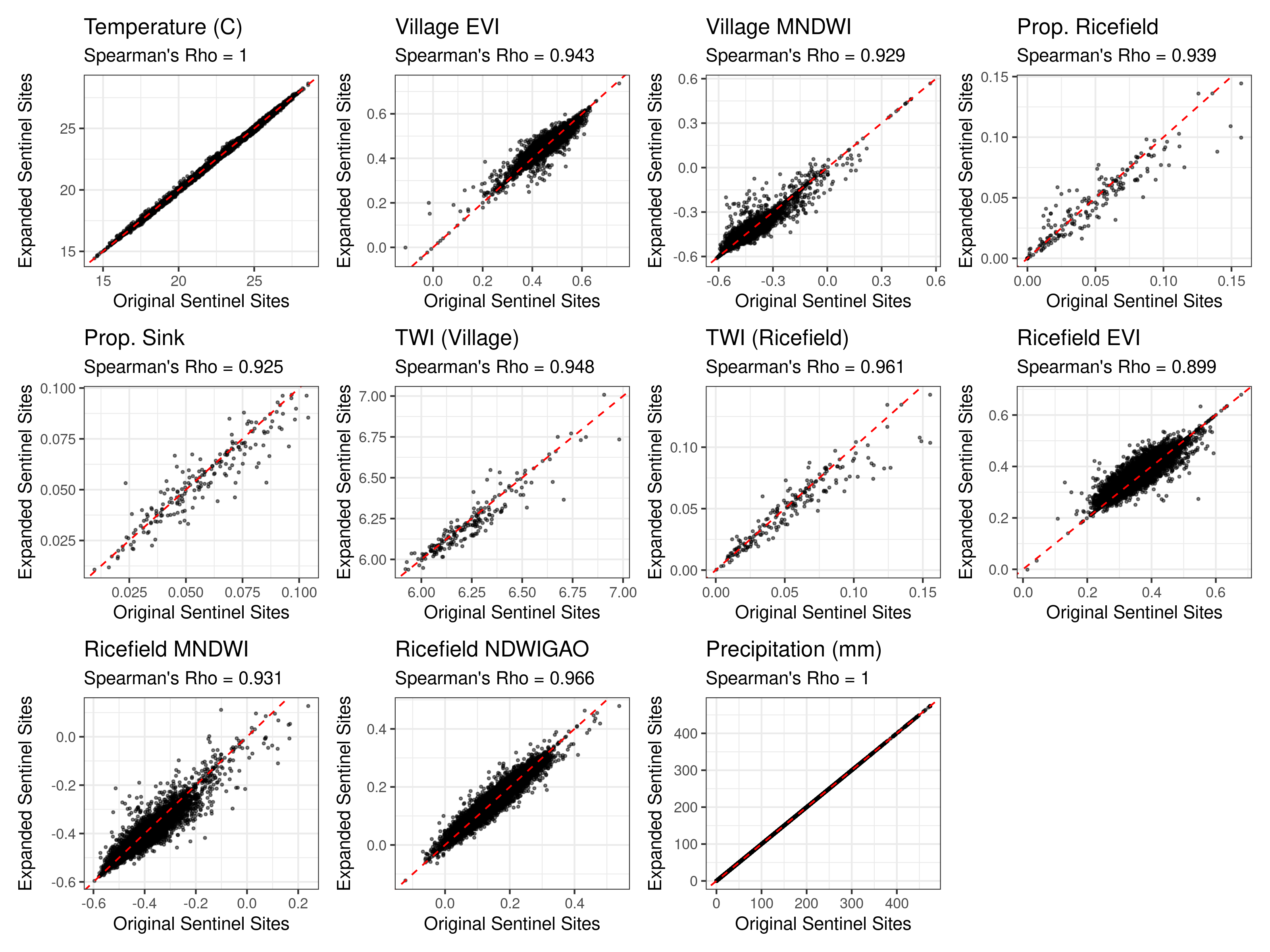


**Figure S1.1.** Scatterplots comparing environmental variables extracted at the original, smaller subset of sentinel village and rice field sites to the expanded dataset. Dynamic variables are a comparison by month and fokontany, while constant variables are only at the fokontany-level. Precipitation data showed no difference due to the coarse resolution of the original data (0.1 degree).

# S2. Creating Rice Indices via Principal Components Analysis

We used principal component analysis to create indices of rice field dynamics from EVI, MNDWI, and GAO-NDWI extracted from rice fields zones. First, we estimated the difference in each indicator at the rice field level from the indicator extracted at a buffer of 1 km surrounding village zones (Δvill-rice) to categorize dynamics specific to rice field environments. Second, we transformed the rice-field level indicators (EVI, MNDWI, and GAO-NDWI) into seasonal anomalies by standardizing each indicator within each calendar month (e.g. January, February, etc.) following Kaul et al. [(2018)](https://www.zotero.org/google-docs/?MUpOQG). This seasonal anomaly represents how conditions differed in that month compared to the same calendar month of different years. Third, we included the original three environmental indicators extracted and average at the major rice field zones. We used all three forms of the three indicators ( Δvill-rice, seasonal anomalies, and original mean; 9 variables total) in the PCA, after having centered and scaled each one. We then selected the first three components, which contained over 73% of the overall variance, to represent three indices of rice field dynamics (Table S1.1, Table S1.2). Rice Index 1 was strongly influenced by all three forms of the MNDWI indicator, and represented the amount of standing water in rice fields. Rice Index 2 was more strongly associated with EVI and NDWI-GAO and represented the vegetation dynamics of the rice fields. Rice Index 3 was most strongly associated with anomalies in the vegetation indices and represented anomalies in vegetation phenology and the timing of the agricultural season.

|  | **PC1** | **PC2** | **PC3** | **PC4** |
| --- | --- | --- | --- | --- |
| **Standard Deviation** | 1.708 | 1.560 | 1.086 | 0.921 |
| **Proportion of Variance** | 0.324 | 0.284 | 0.131 | 0.094 |
| **Cumulative Proportion** | 0.324 | 0.609 | 0.740 | 0.834 |

**Table S2.1.** Proportion of variance explained by the first four components of the PCA used to create the rice indices.

|  | **Dim. 1** | **Dim. 2** | **Dim. 3** |
| --- | --- | --- | --- |
| **MNDWI** | 30.81 | 0.21 | 1.05 |
| **EVI** | 0.98 | 31.59 | 3.56 |
| **NDWI-GAO** | 6.94 | 16.82 | 3.11 |
| **ΔMNDWI** | 28.95 | 1.03 | 0.96 |
| **ΔEVI** | 8.17 | 17.55 | 4.59 |
| **ΔGAO** | 9.9 | 10.03 | 14.54 |
| **MNDWI Anomalies** | 10.24 | 0 | 9.63 |
| **EVI Anomalies** | 0.2 | 12.49 | 25.42 |
| **GAO Anomalies** | 3.82 | 10.28 | 37.15 |

**Table S2.2.** Contribution of each variable to the first three components.

|  | **Dim. 1** | **Dim. 2** | **Dim. 3** |
| --- | --- | --- | --- |
| **MNDWI** | 0.948 | -0.073 | -0.111 |
| **EVI** | -0.169 | 0.899 | -0.205 |
| **NDWI-GAO** | 0.45 | 0.656 | -0.192 |
| **ΔMNDWI** | -0.919 | 0.163 | 0.106 |
| **ΔEVI** | 0.488 | -0.67 | 0.233 |
| **ΔGAO** | -0.537 | -0.507 | 0.414 |
| **MNDWI Anomalies** | 0.547 | 0.003 | 0.337 |
| **EVI Anomalies** | -0.076 | 0.565 | 0.548 |
| **GAO Anomalies** | 0.334 | 0.513 | 0.662 |

**Table S2.3** Coordinates of each variable for the first three PCA components.


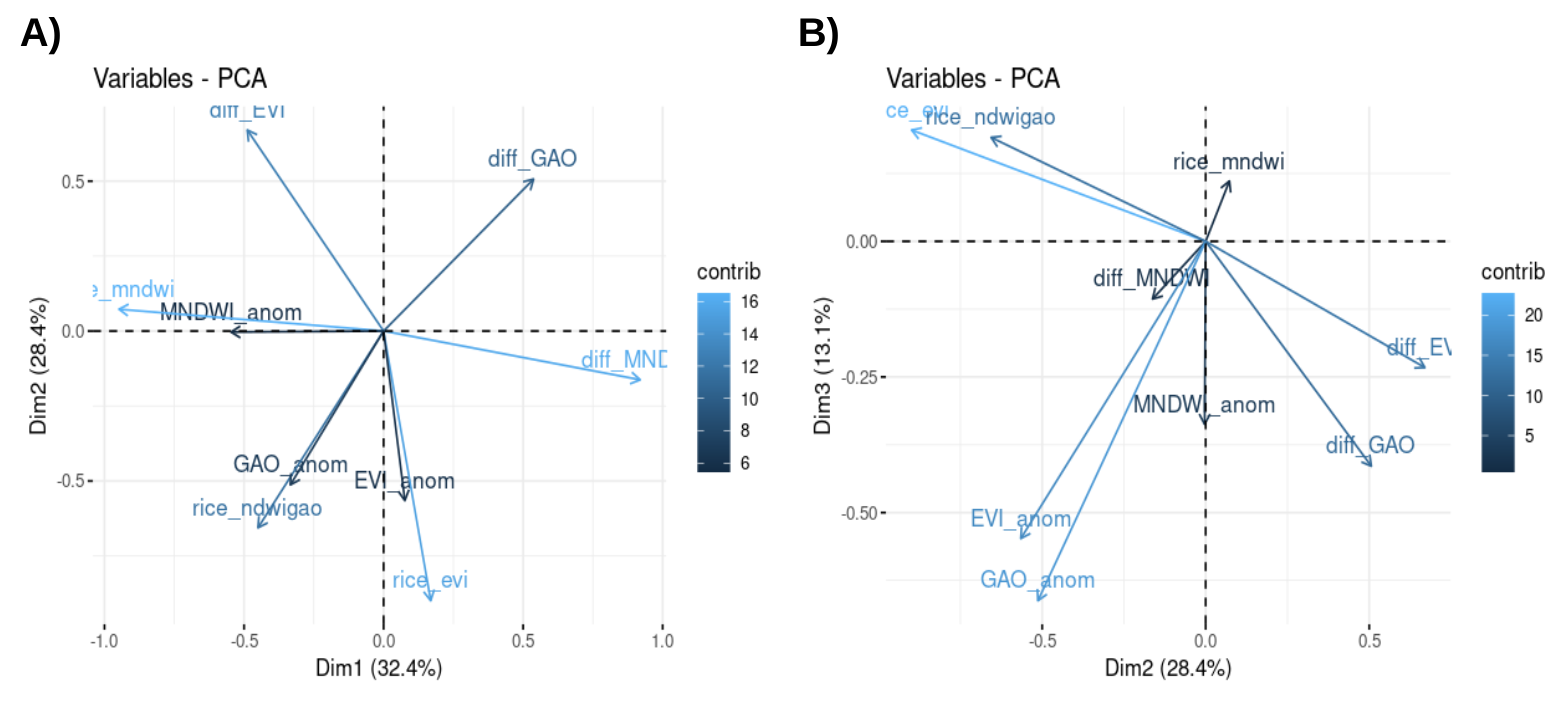


**Figure S2.1.** Plot of PCA Loadings for components 1 and 2 (A) and components 2 and 3 (B).

# S3. Configuring the ZERO-G adjustment for the study zone

#

We applied a zero-adjusted gravity-model estimator (ZERO-G, Evans et al. 2023) to adjust the malaria case data for biases due to geographic barriers, financial barriers, and differing levels of health system support across space and time. We applied the following configurations to the ZERO-G algorithm so that it better conformed to our study system and period:

- Limited the linear trend to be positive, to account for increasing access to the health system over time due to the ongoing health-system strengthening intervention
- Included a “service” of PHCs that represented the COVID-19 pandemic as a binary variable, to account for reduced use of PHCs between March 2020 - July 2021. This allowed the “mass” of a clinic to be reduced during this time period.
- Did not include fokontany missing more than 50% of data or with more than 50% months reporting zero consultations in our estimation of parameters for estimating healthcare access
- Identified zero’s to be imputed via local context. Only those zeros outside of the HSS intervention during the malaria high season (Nov-April) were imputed

# S4. Comparing Non-Linear vs. Linear Model Fit

Many studies have found the relationship between malaria burdens and environmental variables to be non-linear (Okiring et al. 2021, Odhiambo et al. 2020, Zinzser et al. 2012). This is particularly true for climatic variables such as temperature and rainfall, which exhibit unimodal relationships with malaria where malaria burdens decrease at the extremes of the range (Mordecai et al. 2019, Paiijmans et al. 2007). We therefore explored implementing a non-linear model via penalized smoothing splines. Penalized smoothing splines add a penalty to the model to prevent the splines from overfitting the data. We included a penalized smoothing spline for each variable and compared this model to our original linear model described in the main text. We found little difference in model performance or predictions between the two models (Fig. S2.1). Both models had a Spearman’s correlation of 0.64 with the true data and an RMSE (root mean square error) of 61.5. However, the runtime of the non-linear model was nearly 100x longer than the linear model. We therefore chose the more parsimonious linear model for the application.


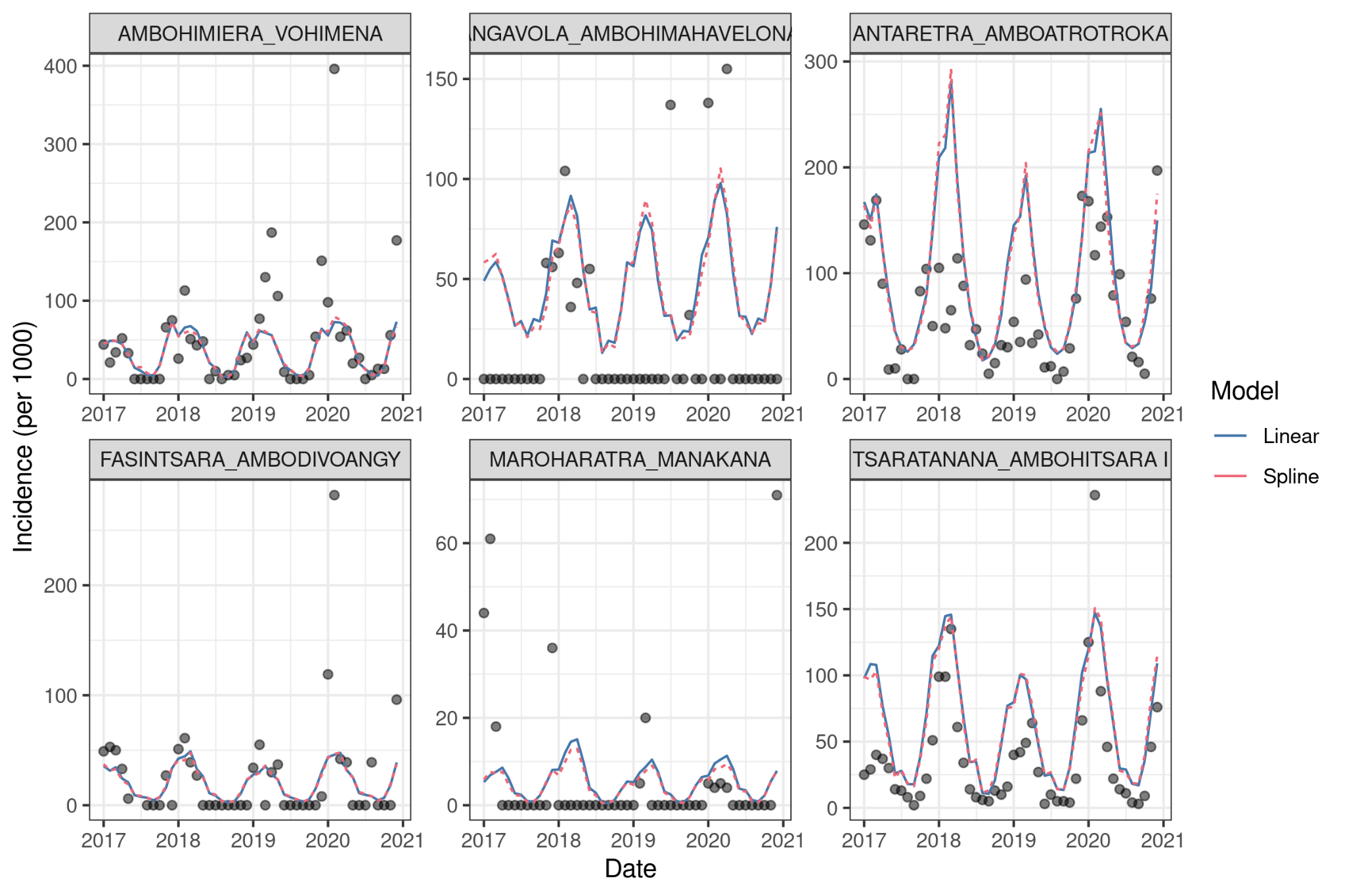


**Figure S4.1. Predictions did not differ between the linear and non-linear penalized spline model.** Predicted incidence rates are plotted for six randomly sampled fokontany, with the true rates shown in black points.

#

# S5. Supplemental Figures


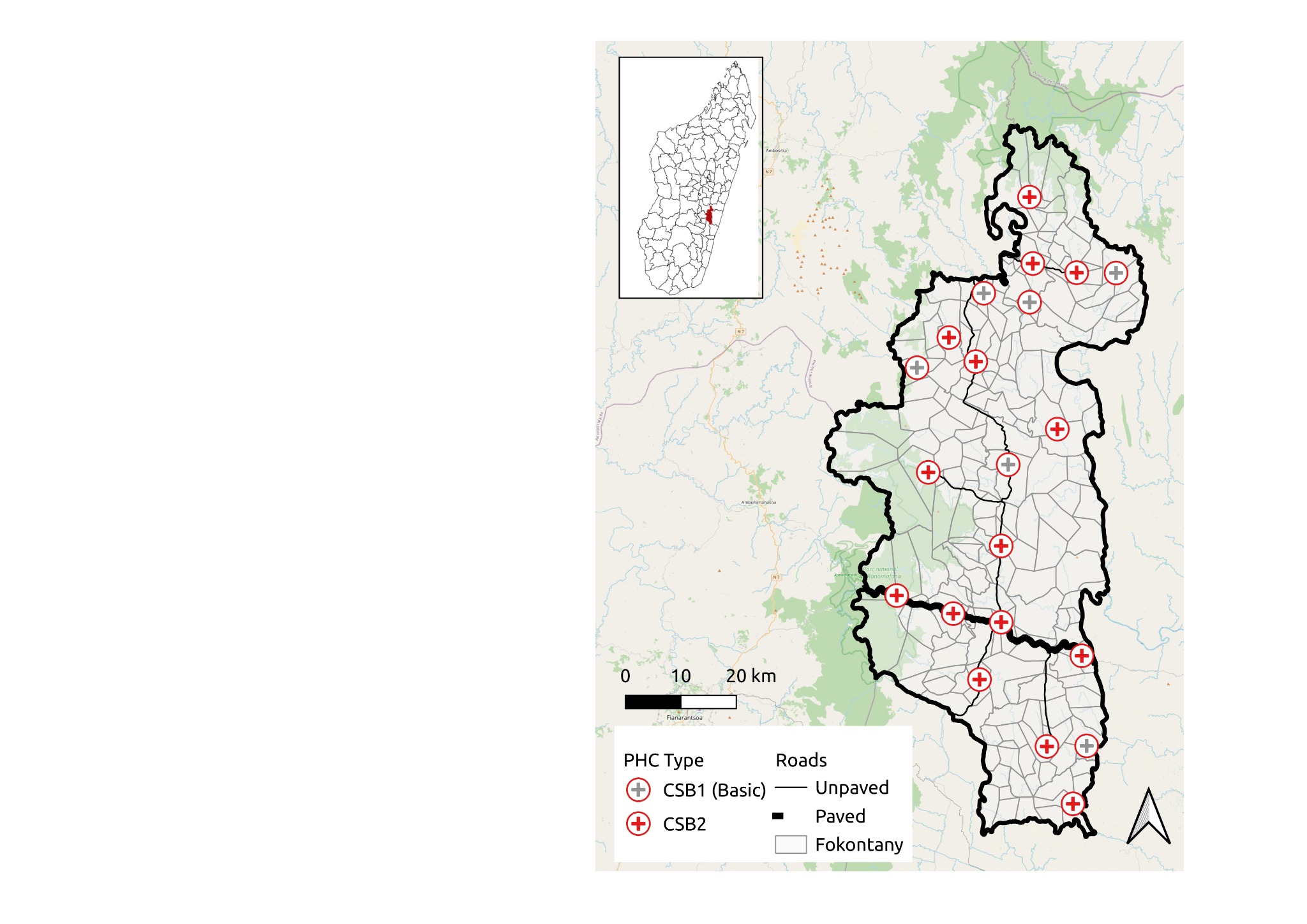


**Figure S5.1. Map of study area (District of Ifanadiana, Vatovavy, Madagascar).** Inset panel locates the district with the country. Background map is sourced from OpenStreetMap.


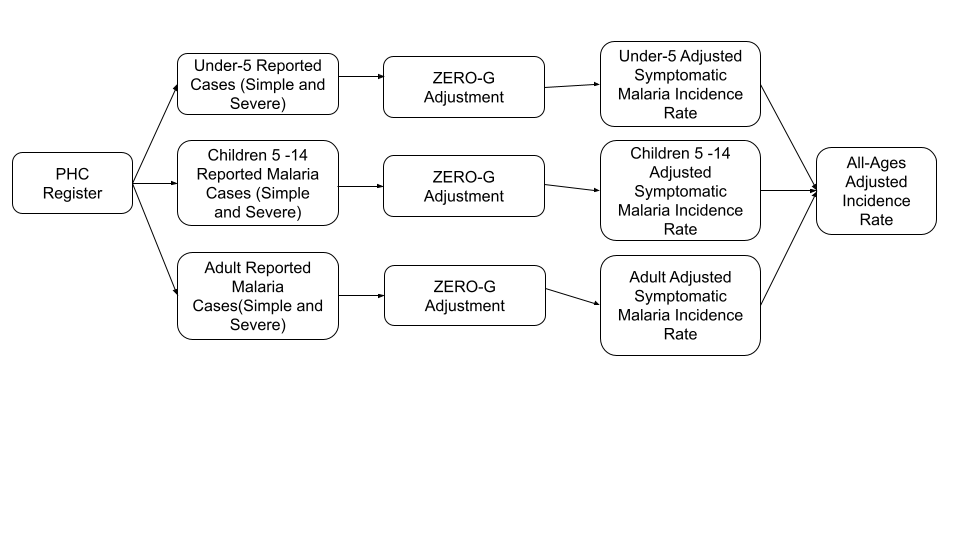
**Figure S5.2. Workflow of data collection and adjustment.** The number of simple and severe malaria cases per month and fokontany are digitized from PHD registers to result in reported cases by age-class. The reported cases are adjusted via the ZERO-G adjustment to result in an age-specific incidence rate of symptomatic cases per month and fokontany. This data is then combined into an all-ages symptomatic malaria incidence rate that is the focus of the forecasting model.


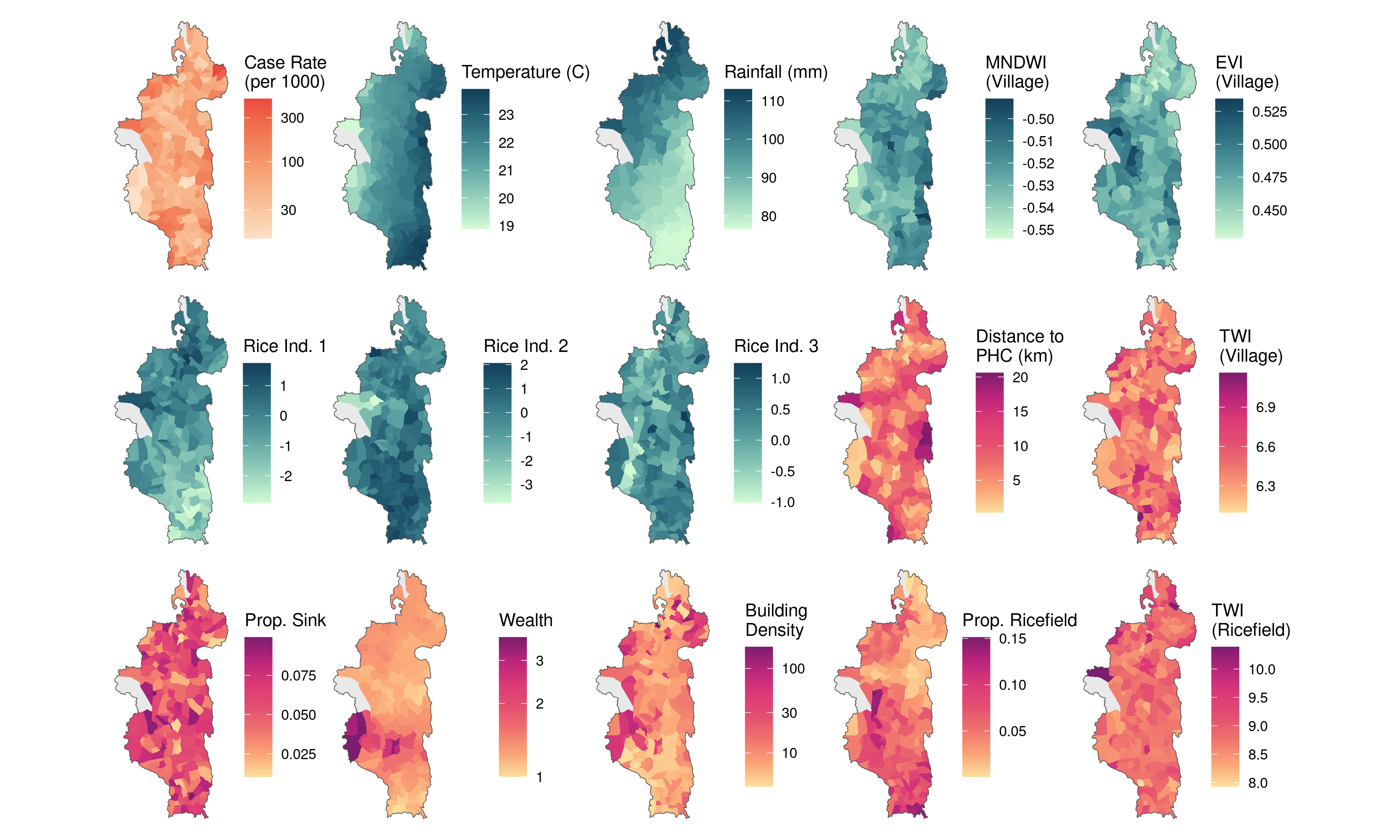


**Figure S5.3. Maps of ZERO-G adjusted malaria case rates and covariates used in the forecasting model at the fokontany level.** Dynamic variables are colored in teal and the average value during the year 2020 is shown. Static variables are plotted in red.


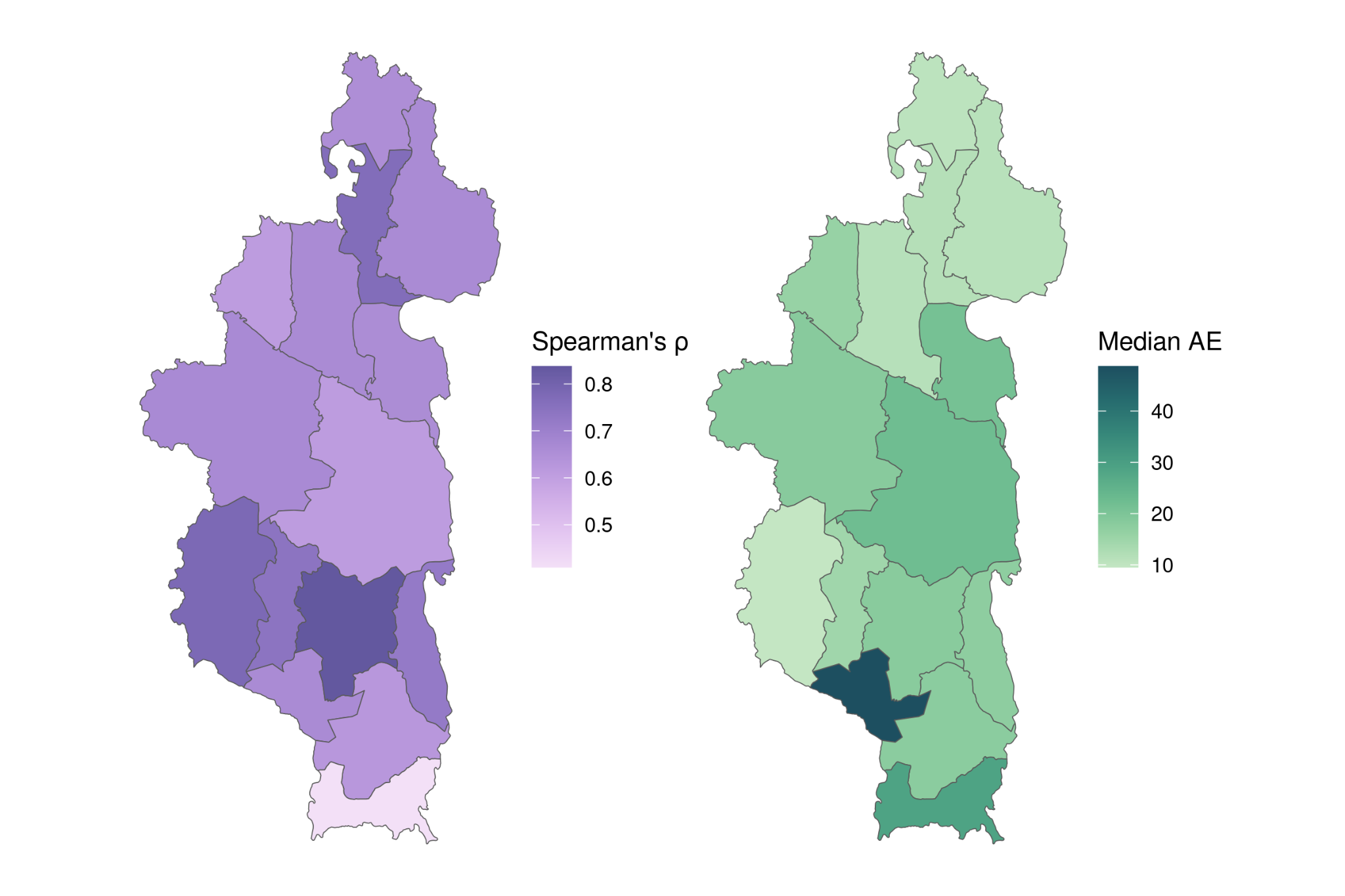


**Figure S5.4. Performance metrics by each spatial block (commune) used as out-of-sample data in the cross-validation procedure.** Median AE represents the median absolute error of between the predicted and observed incidence rates when each commune was out-of-sample, with lower error rates representing higher accuracy.


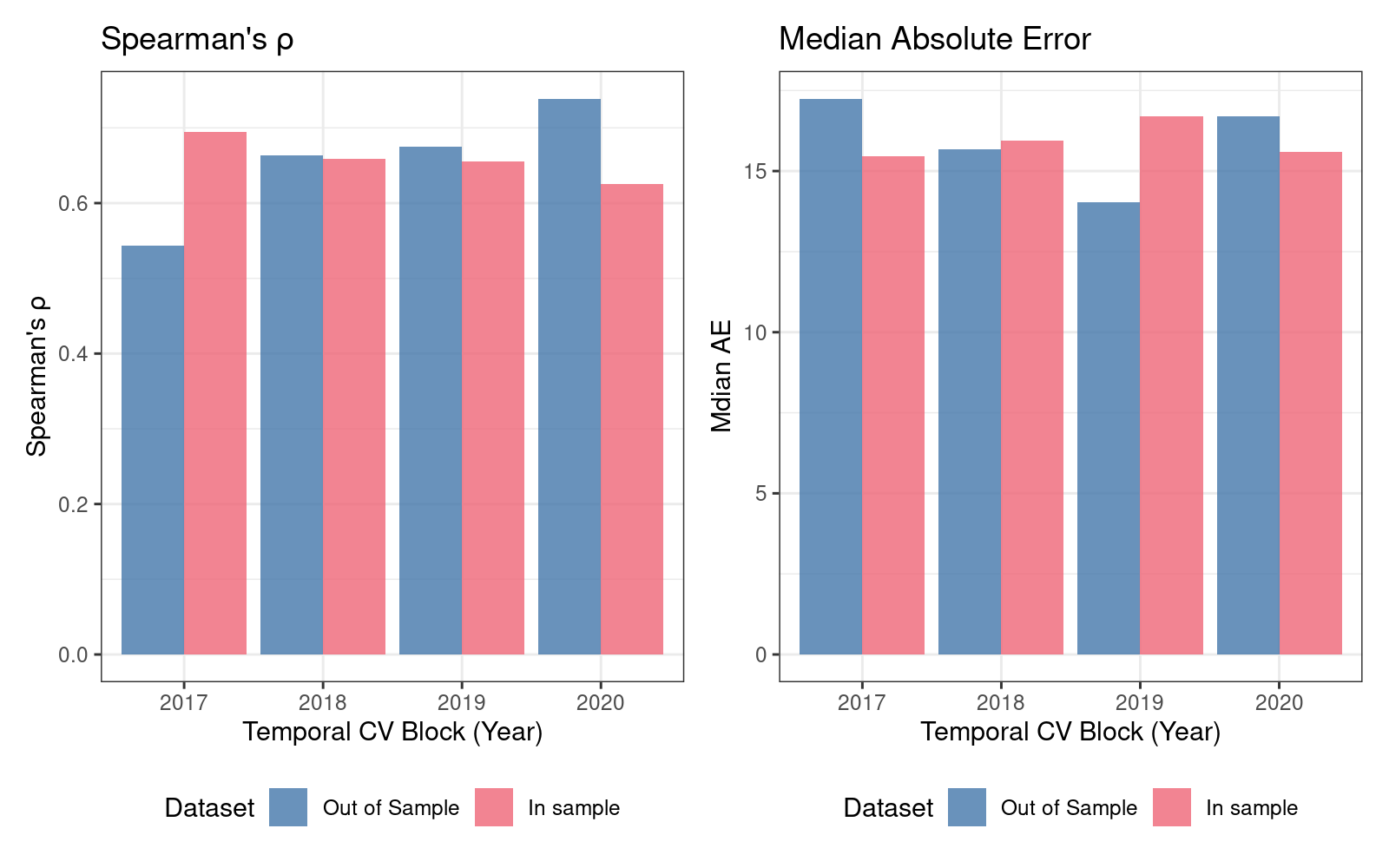


**Figure S5.5. Performance of the model applied to temporal cross-validation by year, with each year representing an out-of-sample cross-validation block.**  Median AE represents the median absolute error of between the predicted and observed incidence rates, with lower error rates representing higher accuracy.


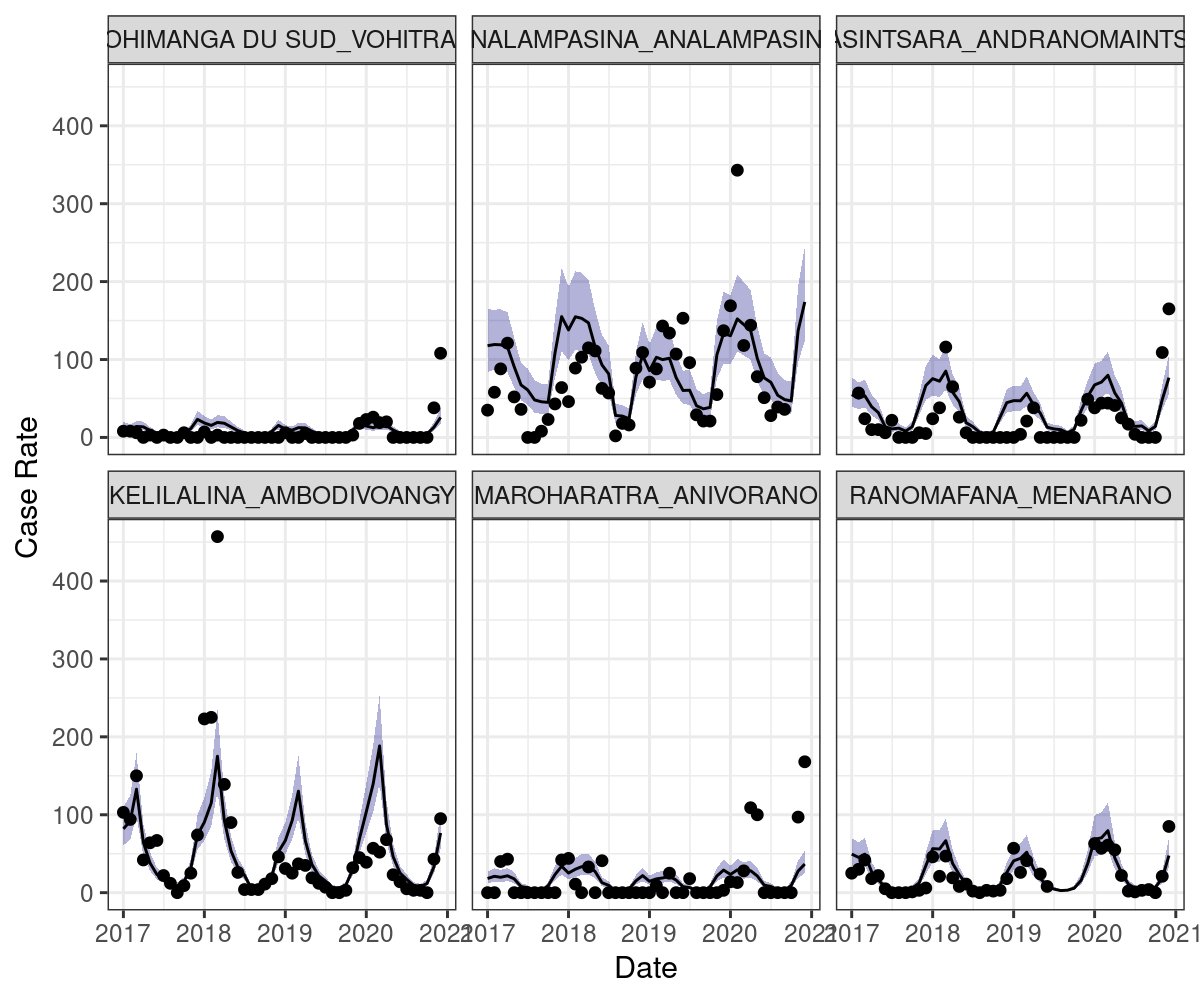


**Figure S5.6. Comparison of SMALLER predictions (line) and estimated case rate per 1000 (points) for six example fokontany.**

# S6. Supplemental Tables

**Table S6.1. Coefficients of fixed effects in full INLA model.**

| **Variable** | **Lower 95% CI** | **Median** | **Upper 95% CI** |
| --- | --- | --- | --- |
| Months since LLIN Distribution | 1.285 | 1.321 | 1.359 |
| Proactive CHW | 0.922 | 1.147 | 1.427 |
| Proportion Ricefield | 0.934 | 1.039 | 1.156 |
| Proportion Sink | 0.931 | 1.027 | 1.134 |
| TWI (Ricefield) | 0.958 | 1.055 | 1.162 |
| TWI (Village) | 0.973 | 1.06 | 1.154 |
| Wealth | 0.729 | 0.808 | 0.895 |
| Building Density | 0.911 | 0.991 | 1.078 |
| Temperature (C) | 1.268 | 1.384 | 1.511 |
| Rainfall (mm) | 1.001 | 1.042 | 1.084 |
| MNDWI (Village) | 1.078 | 1.118 | 1.16 |
| EVI (Village) | 1.007 | 1.061 | 1.118 |
| Rice Index 1 | 0.981 | 1.018 | 1.057 |
| Rice Index 2 | 0.913 | 0.946 | 0.981 |
| Rice Index 3 | 1.012 | 1.052 | 1.093 |

7. Résumé Français

**Contexte :** La disponibilité croissante des données électroniques des systèmes de santé et des variables environnementales télédétectées a conduit à la prolifération de modèles statistiques capables de produire des prévisions sur le paludisme. Nombre de ces modèles ont été mis en œuvre dans des systèmes d'alerte précoce du paludisme (SAPPs), qui fournissent des prévisions sur les dynamiques du paludisme plusieurs mois à l'avance aux niveaux national et régional. Cependant, les systèmes d'alerte précoce ne tirent généralement pas parti des données provenant des villages ou des systèmes de santé communautaires, le premier point de contact pour la majorité des populations rurales dans les pays en développement, pour prévoir le paludisme à des échelles spatiales fines.

**Méthodes :** Nous avons mis au point un système d'alerte précoce hyperlocal destiné à être utilisé dans le cadre d'une intervention de renforcement du système de santé dans les zones rurales de Madagascar. Il combine des données de notification de cas au niveau du village, corrigées des biais, avec des variables environnementales télédétectées à des échelles spatiales aussi fines qu'une résolution de 10 mètres. Un modèle de régression linéaire généralisée hiérarchique spatio-temporel a été entraîné sur des données mensuelles de cas de paludisme provenant de 195 communautés de 2017 à 2020 et évalué par validation croisée. Le modèle a ensuite été intégré dans un flux de travail automatisé avec des données environnementales mises à jour mensuellement pour créer un SAPP à mise à jour continue capable de prédire les cas de paludisme jusqu'à trois mois à l'avance au niveau du village. Les prévisions ont été transformées en indicateurs pertinents pour les acteurs du système de santé en estimant les quantités de fournitures médicales nécessaires à chaque dispensaire et le nombre de cas restant au niveau de la communauté.

**Résultats :** Le modèle statistique a été en mesure de reproduire avec précision les données relatives aux cas survenus dans les villages, avec des performances près de cinq fois supérieures à celles d'un modèle nul au cours de la validation croisée. Les variables environnementales dynamiques, en particulier celles associées aux eaux stagnantes et à la dynamique des rizières, ont été fortement associées à l'incidence du paludisme, ce qui a permis au modèle de prédire avec précision les taux d'incidence futurs. Le SAPP représente une amélioration de plus de 50 % par rapport aux méthodes existantes de quantification des stocks lorsqu'il est appliqué rétrospectivement.

**Conclusion :** Nous avons démontré la faisabilité du développement d'un système d'alerte précoce automatique et hyper-local utilisant des données environnementales télédétectées à des échelles spatiales fines. Les données du système de santé étant de plus en plus numérisées, cette méthode peut être facilement appliquée à d'autres régions et être mise à jour avec des données de santé en temps quasi réel afin d'améliorer encore les performances.

S8. References

Kaul, R. B., M. V. Evans, C. C. Murdock, and J. M. Drake. 2018. Spatio-temporal spillover risk of yellow fever in Brazil. Parasites & Vectors 11:488.

Randriamihaja, M., F. A. Ihantamalala, F. H. Rafenoarimalala, K. E. Finnegan, L. Rakotonirina, B. Razafinjato, M. H. Bonds, M. V. Evans, and A. Garchitorena. 2024. Combining OpenStreetMap mapping and route optimization algorithms to inform the delivery of community health interventions at the last mile. PLOS Digital Health 3:e0000621.
